# Effective protection of photoreceptors using an inflammation-responsive hydrogel to attenuate outer retinal degeneration

**DOI:** 10.1038/s41536-023-00342-y

**Published:** 2023-12-14

**Authors:** Hyerim Kim, Hyeonhee Roh, Sang-Heon Kim, Kangwon Lee, Maesoon Im, Seung Ja Oh

**Affiliations:** 1 Program in Nanoscience and Technology, Graduate School of Convergence Science and Technology, Seoul National University, Seoul, 08826 South Korea; 2 Brain Science Institute, Korea Institute of Science and Technology (KIST), Seoul, 02792 South Korea; 3 School of Electrical Engineering, College of Engineering, Korea University, Seoul, 02841 South Korea; 4 Center for Biomaterials, Biomedical Research Institute, KIST, Seoul, 02792 South Korea; 5 Division of Bio-Medical Science and Technology, KIST School, University of Science and Technology (UST), Seoul, 02792 South Korea; 6 Department of Applied Bioengineering, Graduate School of Convergence Science and Technology, Seoul National University, Seoul, 08826 South Korea; 7 Research Institute for Convergence Science, Seoul National University, Seoul, 08826 South Korea; 8 KHU-KIST Department of Converging Science and Technology, Kyung Hee University, Seoul, 02447 South Korea; 9 Department of Genetics and Biotechnology, College of Life Sciences, Kyung Hee University, Yongin, 17104 South Korea

**Keywords:** Retina, Cell death in the nervous system

## Abstract

Retinitis pigmentosa (RP) is an outer retinal degenerative disease that can lead to photoreceptor cell death and profound vision loss. Although effective regulation of intraretinal inflammation can slow down the progression of the disease, an efficient anti-inflammatory treatment strategy is still lacking. This study reports the fabrication of a hyaluronic acid-based inflammation-responsive hydrogel (IRH) and its epigenetic regulation effects on retinal degeneration. The injectable IRH was designed to respond to cathepsin overexpression in an inflammatory environment. The epigenetic drug, the enhancer of zeste homolog 2 (EZH2) inhibitors, was loaded into the hydrogel to attenuate inflammatory factors. On-demand anti-inflammatory effects of microglia cells via the drug-loaded IRH were verified in vitro and in vivo retinal degeneration 10 (*rd10*) mice model. Therefore, our IRH not only reduced intraretinal inflammation but also protected photoreceptors morphologically and functionally. Our results suggest the IRH reported here can be used to considerably delay vision loss caused by RP.

## Introduction

Retinitis pigmentosa (RP) is an outer retinal degenerative disease which progressively damages rod and cone photoreceptor cells, resulting in profound vision loss. At the early stage of this ailment, the gradual death of rod cells is known to happen due to mutations in any of more than 90 different genes^1^. After the rod degeneration, cone cell death is followed by microenvironmental changes including retinal oxidative stress and inflammation^2^. Particularly, inflammation is also known to be associated with retinal degeneration itself^3^: Activated inflammatory microglia exhibit phagocytic engulfment of non-apoptotic rods and secrete inflammatory molecules, eventually accelerating both rod apoptosis and cone degeneration^4–6^. Therefore, anti-inflammatory treatments on inflammatory microglia may substantially delay the progression of retinal degeneration, potentially saving photoreceptors from cell death. Recent studies have demonstrated that epigenetic regulation of the degenerative retinal microenvironment improves cone cell survival^4^ and suppresses inflammation in an RP animal model^7^. In particular, it has been reported that enhancer of zeste homolog 2 (EZH2) is overexpressed in the degenerate retina of a mouse model, contributing to rod cell death^8^. Also, the inhibition of EZH2 effectively attenuated level of inflammatory microglia in a neuroinflammatory disease^9^. Therefore, the suppression of EZH2 may potentially have similar anti-inflammatory effect in retinal degenerative diseases such as RP; however, it has not been studied as a therapeutic approach for any of retinal degenerative diseases.

Anti-inflammatory drugs can be intravitreally administered into the intraocular space. However, the intraocular injection of drugs has some limitations such as fast drug elimination via anterior or posterior clearance, non-specific distribution, and need for repeated injections^10^. These issues can be addressed by the use of an appropriate drug carrier for intraocular administration, but its development has been challenging because eye is a highly complexed and specialized organ of the body. As a major component of vitreous humor, hyaluronic acid (HA) has been widely studied as a drug carrier for eye diseases (e.g., glaucoma and dry eye disease) due to its high biocompatibility, biodegradability, viscoelasticity, and injectability^11^. Even with those benefits, HA still has a couple of limitations: a short retention time in the intraocular tissue and uncontrolled manner of drug delivery. To overcome these limitations, crosslinked HA has been developed and showed improved retention time, sustained drug release, and higher stability^12^. However, there have been concerns about the toxicity of chemical crosslinking agents (i.e., divinyl sulfone, glutaraldehyde, and carbodiimide), which are used for improving the mechanical properties of HA^13^. Therefore, the crosslinking method based on copper-free click chemistry can be an alternative option for the HA hydrogel since it has advantages such as rapid gelation time, high mechanical properties, and biocompatibility^14,15^. Also, for both enhancing therapeutic effects and attenuating drug side effects, hydrogels releasing drugs in response to diverse stimuli have been developed aiming for eye disease treatment. For example, several hydrogels have demonstrated thermo-sensitive^16^, matrix metalloproteinase-sensitive^17^, and photo-sensitive^18^ characteristics. Although the stimulus-responsive feature is attractive for the accurate on-demand drug delivery, those hydrogels have not been validated for therapeutic effects in retinal degeneration. Moreover, inflammation-responsive function has not been yet implemented.

In the present study, we report an inflammation-responsive syringe-injectable HA-hydrogel which is capable of on-demand release of anti-inflammatory drugs (i.e., EZH2 inhibitor). We have designed the HA-based hydrogel to provide effective intraretinal delivery of EZH2 inhibitor in response to intraocular inflammation, and thereby to induce an anti-inflammatory effect via epigenetic regulation of inflammatory microglia during the progressing retinal degeneration, eventually resulting in delayed vision loss. To assess the responsive function of the inflammation-responsive hydrogel (IRH), we have investigated drug release kinetics in response to cathepsins which are known to be overexpressed in inflammatory environments. Then, we have evaluated the anti-inflammatory effect of the EZH2 inhibitor-loading hydrogel in vitro using inflammatory SV40 microglia cell line. We have also confirmed the performance delaying retinal degeneration of the IRH in vivo using immunofluorescence staining of microglia and inflammation markers in the retinal degeneration (*rd10*) mouse retinas, which is an RP mouse model. Lastly, the alleviated loss of visual function has been demonstrated via electrophysiological recordings of retinal ganglion cells, which are retinal output neurons, indicating more photoreceptors were saved from the degeneration than those in the control group.

## Results

### Cathepsin is overexpressed by inflammation activities of retinal degeneration

**Fig. 1:**
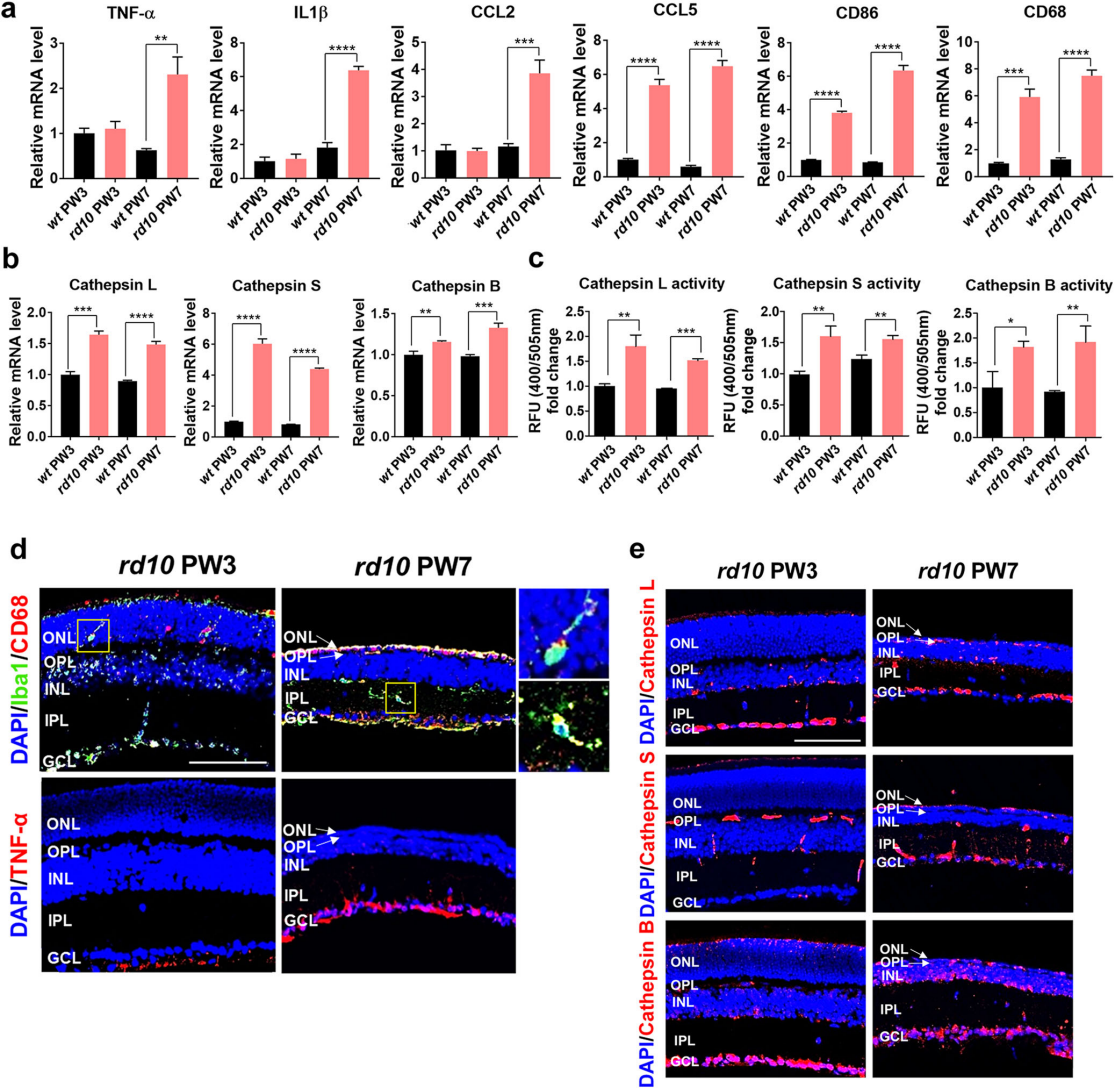
Overexpression of inflammatory factors and cathepsins in retinitis pigmentosa in vivo model. **a** mRNA expression level of inflammatory markers on postnatal week 3 (PW3) and 7 (PW7) after in wild-type (*wt*) and retinal degeneration (*rd10*) mouse model (mean ± SD; *n* = 3). **b** mRNA level of cathepsin L; S; and B at different time points in *wt* and *rd10* mouse model (mean ± SD; *n* = 3). **c** Cathepsins activity in *wt* and *rd10* mouse model (mean ± SD; *n* = 3). Statistical analysis was performed using a one-way analysis of variance (ANOVA) followed by Tukey’s *post hoc* test (*p < 0.1, **p < 0.01, ***p < 0.001, and ****p < 0.0001). **d** Immunofluorescence staining of Iba1 (microglia; green); CD68 (phagocytosis; red); and TNF-α (inflammatory marker; red) in *rd10* mouse model. Magnified views of the two yellow-boxed areas are shown in the right. Inflammatory microglia (Iba1 + CD68+) in the yellow box showed migration from the ONL to the IPL when retinal degeneration progressed and the ONL thinned in PW7 *rd10* mice. **e** Immunofluorescence staining of cathepsin L; S; and B at different time points in the *rd10* mouse model. ONL Outer Nuclear Layer (photoreceptor cell bodies), OPL Outer Plexiform Layer, INL Inner Nuclear Layer (bipolar cell bodies), IPL Inner Plexiform Layer, GCL Ganglion Cell Layer. Scale bar indicates 50 μm and applies to all images.

To identify a target biomarker for inflammation-responsive hydrogel (IRH), we first explored biomolecules which are increased by inflammation as retinal degeneration (RD) progresses. For example, it has been demonstrated that cathepsin enzymes are overexpressed in an inflammatory environment such as skin wound site, joints with rheumatoid, and inflammatory brain disease^19^. High levels of cathepsin are secreted by immune cells in the inflammatory environments such as inflammatory M1 macrophages^20,21^. Cathepsins are cysteine proteases which are key players in physiological processes of various inflammatory diseases^22^. In addition, the secreted cathepsins degrade the extracellular matrix, and the proteolytic ability of cathepsins enables the development of a cathepsin-sensitive hydrogel through the generation of a protein crosslinker degraded by cathepsins. As diverse cathepsin subtypes are oversecreted in the inflammatory microenvironment simultaneously^23^, we designed a cathepsins-responsive hydrogel to respond to various cathepsin enzymes. To test whether the cathepsin can serve as a biomarker for IRH in RD, we analyzed the levels of several inflammatory markers and cathepsin subtypes in the retinas of the *rd10* mice, a well-established RP model. First, we measured the mRNA expression levels of six representative inflammatory markers: tumor necrosis factor (TNF)-α, interleukin 1β (IL1β), C-C motif chemokine ligand 2 (CCL2), C-C motif chemokine ligand 5 (CCL5), T-lymphocyte activation antigen CD86, and cluster of differentiation 68 (CD68). Animals were sacrificed at postnatal weeks (PW) 3 or 7, which are corresponding to early and intermediate stages of RD, respectively^24–26^; age-matched wild-type (*wt*) mice were also used as control groups. At the PW3, the first three markers (i.e., TNF-α, IL1β, and CCL2) appeared not increasing while the other three markers (i.e., CCL5, CD86, and CD68) showed significantly escalated mRNA levels even from this early RD stage (compare the left-most two bars of subpanels in Fig. 1a). The expression levels of all inflammatory factors we tested were elevated as the disease progressed to the age of PW7 in the *rd10* mice (compare the right-most two bars of subpanels in Fig. 1a). These results clearly show that RP accompanies with inflammation activities.

Relative to the inflammatory response, the mRNA and protein activity levels of the three subtypes (i.e., L, S, and B) of cathepsin enzymes were also upregulated in the *rd10* mice from PW3 (Fig. 1b, c). In addition to the mRNA level analyses and the protein activity assays, we further performed immunofluorescence staining of the aforementioned biomolecules. In particular, CD68 as a phagocytic function of inflammatory microglia (Iba1 + CD68+) in the retina, which is known to accelerate photoreceptor degeneration^4^, was detected in the outer nuclear layer (ONL) and the inner plexiform layer (IPL) at PW3 and PW7 of *rd10* animals, respectively (top row of Fig. 1d). Also, the other inflammatory marker TNF-α was found in the ganglion cell layer (GCL) (bottom row of Fig. 1d) and it was consistent with the mRNA level results that PW7 animal showed increased level (compare with the left-most subpanel of Fig. 1a). Interestingly, cathepsins were observed throughout more retinal layers in the immunofluorescence staining results (Fig. 1e): for example, all subtypes of cathepsins were weakly shown in outer segments of photoreceptors (i.e., just above the ONL) at PW3 of animals (the left column of Fig. 1e). Strong bands of cathepsins L and B were also found at that early stage of RD. Then, the expression of all three subtypes seems widely spreading as the RD advances (the right column of Fig. 1e). Collectively, the inflammatory environment induced by inherited RD resulted in cathepsin overexpression. These results indicate that targeting cathepsins enables to development of inflammatory response hydrogels, which can also be applied to the treatment of RP.

### Fabrication of injectable inflammation-responsive hydrogel that enables on-demand anti-inflammatory drug release depending on disease activity

**Fig. 2:**
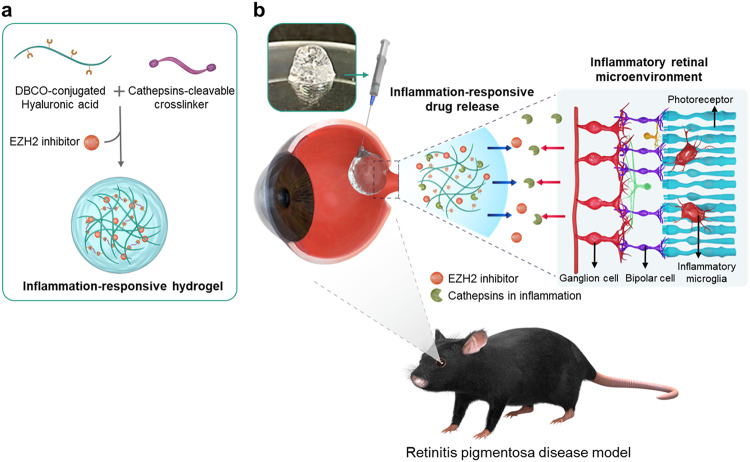
Schematic illustration of syringe-injectable inflammation-responsive hydrogel for suppression of inflammatory microglia for preventing photoreceptor death in retinitis pigmentosa. **a** Inflammation-responsive hydrogel is formed by crosslinking of the DBCO conjugated with hyaluronic acid and azide of cathepsins-cleavable crosslinker. **b** The EZH2 inhibitor containing hydrogel response to cathepsins overexpressed in inflammation environment of the retina; and suppress the inflammation via disease-dependent released EZH2 inhibitor from the hydrogel.

It has been demonstrated that on-demand drug delivery can not only reduce the side effects of overdose but also enhance the therapeutic effects by optimizing the amount of drug delivered depending on the severity of the target disease^17,23,27^. We fabricated IRHs via crosslinking between DBCO-conjugated HA and cathepsin-cleavable crosslinker. Also, EZH2 inhibitor was encapsulated when hydrogel was crosslinked (Fig. 2a). The decomposition rate of the IRH was designed to be dependent on the amount of cathepsin secreted in the inflammatory microenvironment. The fabricated hydrogel containing the drug was intravitreally injectable into eyeball (Fig. 2b). The IRH was disassembled by oversecreted cathepsins in the inflammatory retinal microenvironment and released a pre-loaded EZH2 inhibitor. Therefore, the hydrogel effectively mitigated inflammation by the on-demand delivery of an EZH2 inhibitor in response to inflammatory activities arising from the early stage of RP.

**Fig. 3:**
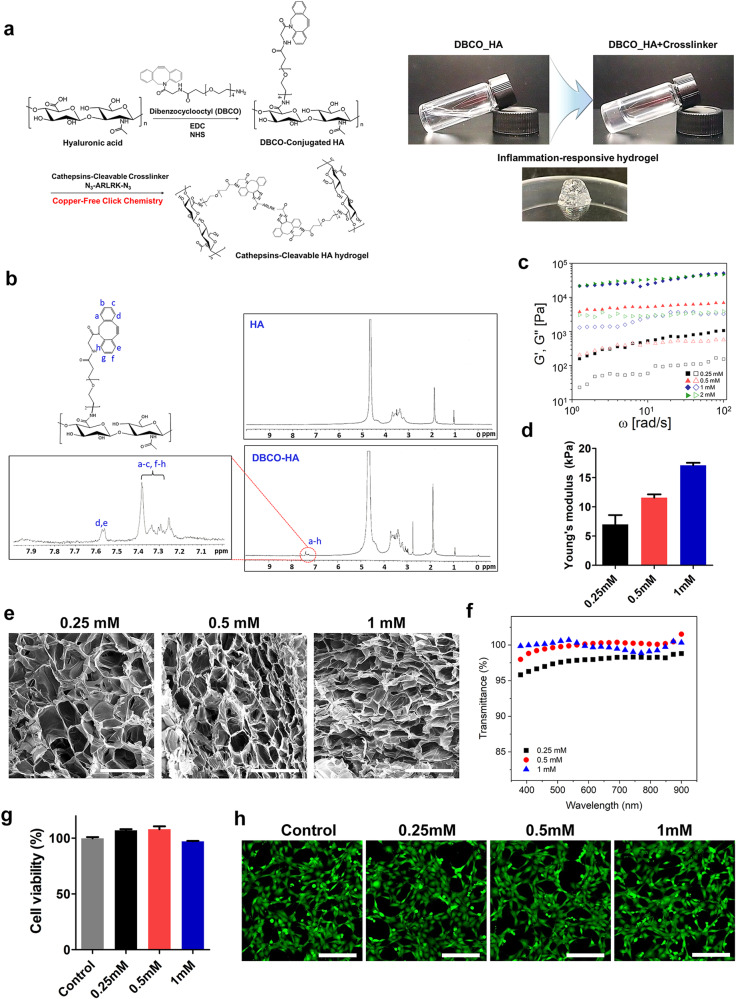
Characterization of injectable inflammation-responsive hydrogel. **a** Fabrication steps of HA-based inflammation-responsive hydrogel (IRH). **b** Chemical composition analysis by ^1^H-NMR. **c** Storage modulus (G’; solid symbols) and loss modulus (G”; hollow symbols) of IRHs made with four different concentrations (i.e.; 0.25; 0.5; 1; and 2 mM) of cathepsins-cleavable crosslinker. **d** Stiffness of hydrogels via Young’s modulus value (mean ± SD; *n* = 3). **e** Scanning electron microscope (SEM) images of hydrogel network. Scale bar indicates 200 μm and applies to all images. **f** Transmittance values of IRHs are plotted as a function of light wavelength. **g** Cell viability of fibroblasts cultured with hydrogels. The cultured dish was incubated with CCK-8 solution; and the supernatant was harvested and measured absorbance at 450 nm (mean ± SD; *n* = 3). **h** After the 24-hour-long culture with IRH; fibroblasts were stained using Cytotoxicity Assay Kit. Live cells are shown in green while dead cells are not visible; which are supposed to be in red. Scale bar indicates 200 μm and applies to all images.

An injectable IRH was fabricated by copper-free click chemistry between DBCO of HA and azide of cathepsin-cleavable crosslinker (c-c crosslinker) (Fig. 3a). The successful conjugation of DBCO to HA was confirmed using ^1^H-NMR (*see* Experimental Section): multiple peaks indicating DBCO were detected between 7 and 8 ppm (*see* enlarged inset shown in left of Fig. 3b), which is consistent with previous studies^14,28^. Also, the mechanical properties were analyzed with a rheometer, and as the c-c crosslinker increased, both storage modulus (G’) and loss modulus (G”) increased but were saturated at a concentration of 2 mM (Fig. 3c). Since storage modulus (G’) represents elasticity and loss modulus (G”) represents the viscosity of a material, an increase in both parameters means that the viscoelasticity of the IRH accelerates. Stiffness of the IRH, which was measured by Young’s modulus, appeared increasing with the higher concentration of the c-c crosslinker and the modulus was ~18 kPa in average when 1 mM c-c crosslinker was used (Fig. 3d). Given the low modulus of vitreous humor (typically known to be several Pa but varies depending on studies), there may be a possibility of adverse effect(s) stemming from a mechanical mismatch between the hydrogel and the vitreous, which need to be studied in future researches. The internal network structure of the hydrogel became denser as the c-c crosslinker concentration increased as shown in scanning electron microscope (SEM) images (Fig. 3e). A denser internal network means a slower rate of biodegradation, extending the residence time of the hydrogel in the disease site. Since the most important property of materials for intraocular injection is transparency, we measured transmittance of the fabricated IRH; the transparency was 98–100% for all the c-c crosslinker concentrations we tested (i.e., 0.25, 0.5, and 1 mM), indicative of no hindrance to remaining vision of RP subjects when the IRH is implanted in the future clinical use (Fig. 3f). Lastly, the in vitro cytotoxicity of the IRH was investigated using cell viability assay and Live & Dead/Cytotoxicity Assay Kit (#L3224, Invitrogen, Waltham, Massachusetts, U.S.A.); our results clearly demonstrated that the hydrogel was non-toxic for the several c-c crosslinker concentrations (Fig. 3g, h). All of these results indicate that our IRHs have appropriate mechanical, optical, and biocompatible characteristics for intraocular implantation.

### On-demand drug release in response to inflammation-related enzymes

**Fig. 4:**
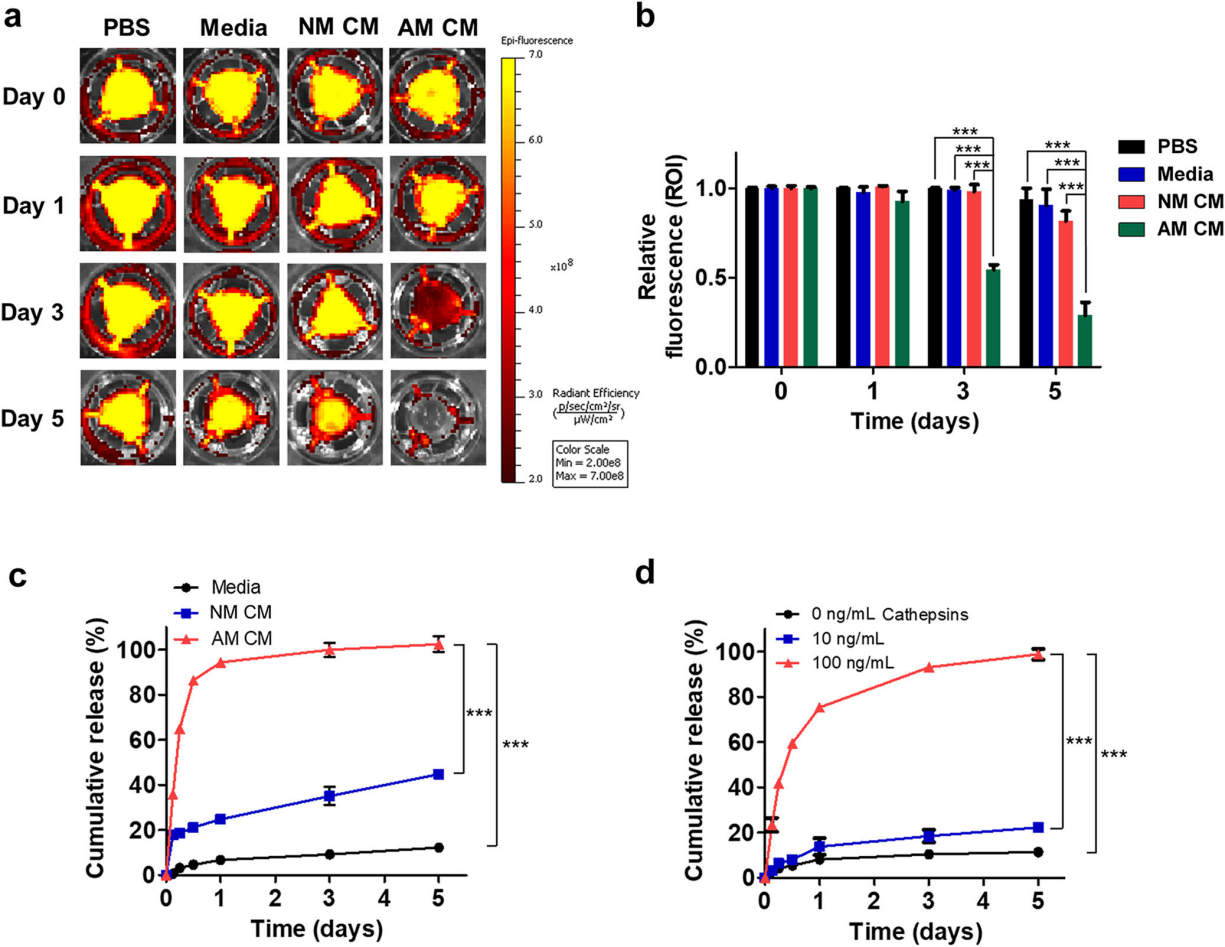
EZH2 inhibitor release amplified in response to the inflammatory environment. **a** Dextran-loaded hydrogels were incubated in PBS; control media; non-activated microglia conditioned media (NM CM); and activated microglia conditioned media (AM CM) and imaged by in vivo imaging systems (IVIS). **b** Relative fluorescence intensity (Region of interest; ROI) of remained dextran at each time point (mean ± SD; *n* = 3). **c** EZH2 inhibitor cumulative release profile from the hydrogel in control media; NM CM and AM CM. The supernatant containing EZH2 was measured at room temperature using UV absorbance at 256 nm (mean ± SD; *n* = 3). Data points within Day 1 show the short-term release profile. **d** In vitro release kinetics of EZH2 inhibitor from the hydrogel in PBS at 37 °C with three different cathepsins concentrations (i.e.; 0; 10; and 100 ng/mL) (mean ± SD; *n* = 3). Statistical analysis was performed using a one-way analysis of variance (ANOVA) followed by Tukey’s *post hoc* test (***p < 0.001).

To test the inflammatory responsiveness of the hydrogel, dextran fluorescence dye and small molecule-encapsulated hydrogels were incubated with inflammatory microglia conditioned media and cathepsins. Dextran release from the hydrogels fabricated with an identical condition (c-c crosslinker concentration of 1 mM and some other condition parameters) was maintained for 5 days in four different solutions: a phosphate buffered solution (PBS), control media, non-activated microglia conditioned media (NM CM), and activated microglia conditioned media (AM CM). Therefore, we hypothesized that AM CM reflects the inflammatory microenvironment of RP in which inflammatory microglia are present. The amounts of dextran release in each hydrogel were visualized using in vivo imaging systems (IVIS) (Fig. 4a) and summarized in a form of bar graphs (Fig. 4b). As shown in both plots (Fig. 4a, b), the IRHs showed rapid release characteristics in the activated microglia media. The dextran intensity was significantly decreased in the AM CM group at day 3 (0.55 ± 0.03) and day 5 (0.29 ± 0.07), whereas the NM CM group did not show a distinct decrease at day 3 (0.98 ± 0.04) and day 5 (0.82 ± 0.06). These results indicate that the dye is rapidly released by the disassembled network of IRH in the inflammatory microenvironment. The small molecule EZH2 inhibitor-encapsulated hydrogel was incubated with a conditioned medium, which is a medium for microglia cultured for 1 day. Consistent with the IVIS results (Fig. 4a), the release rate of the EZH2 inhibitor was accelerated in the activated microglia conditioned media (AM CM) (Fig. 4c), suggesting the possibility of on-demand drug release depending on the inflammatory severity. However, the EZH2 inhibitor showed a more rapid release rate than the dextran dye because of its small molecular weight. In addition, a related inflammatory enzyme (i.e., cathepsin L, S, and B) increased the release of EZH2 inhibitors with increasing cathepsin concentration (Fig. 4d). In the control group without cathepsins, only ~10% of EZH2 inhibitor was released for 5 days while 10 ng/mL of cathepsins induced ~20% of EZH2 inhibitor release. Furthermore, in the case of 100 ng/mL of cathepsins, ~80% of EZH2 inhibitor was released on the first day and reached 100% release on day 5. Taken together, the IRHs exhibited active drug release capabilities in response to inflammatory conditions. Probably, repeated injection of EZH2 inhibitor with IRH might be needed in clinical cases depending on the recurrence of inflammations; but the injection frequency may be substantially lowered due to the inflammation-responsive nature of our hydrogel.

### Inflammatory microglia are attenuated by EZH2 inhibitor-loaded hydrogel

**Fig. 5:**
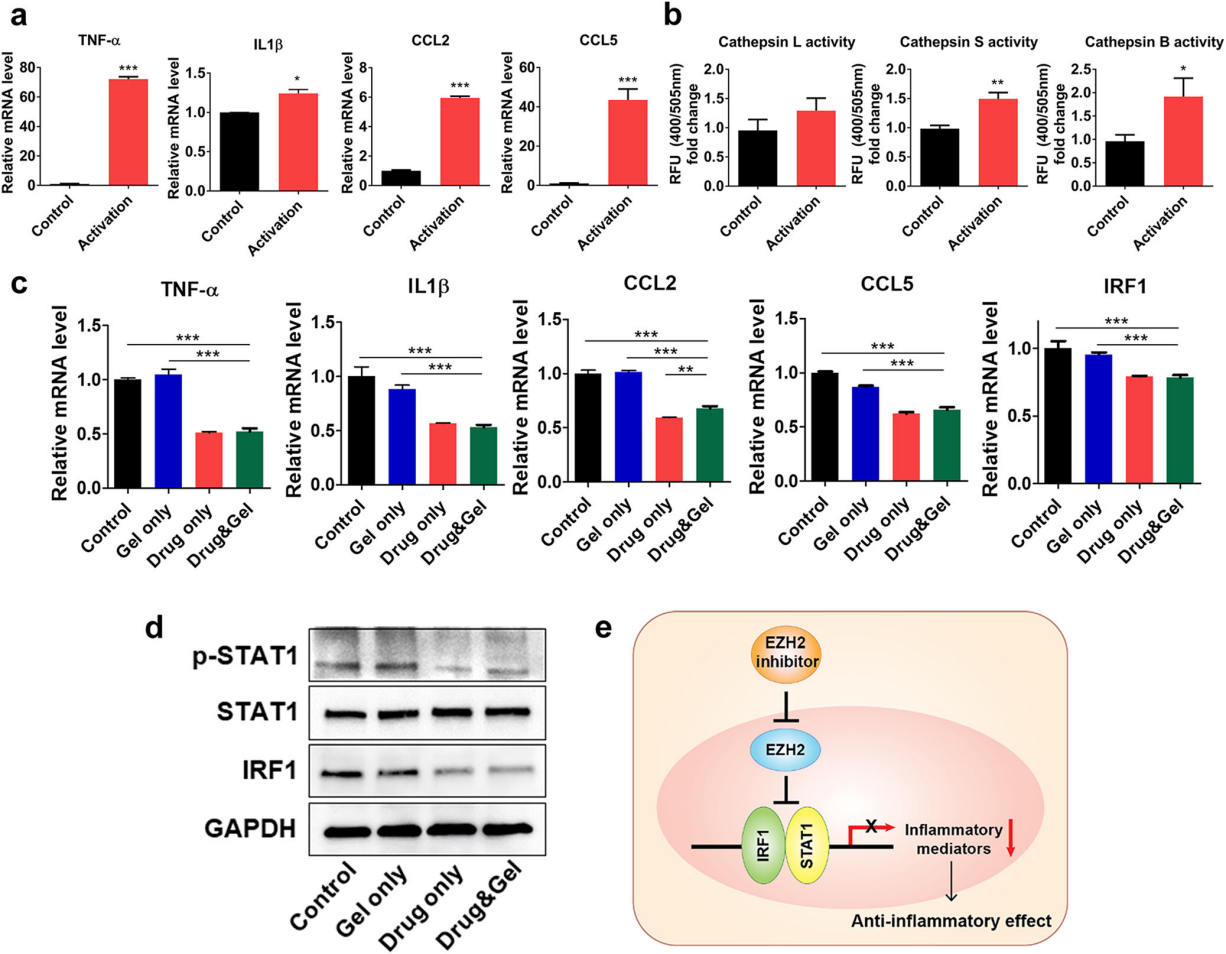
EZH2 inhibitor-loaded hydrogel for anti-inflammatory effect via epigenetic modification. **a** mRNA level of inflammatory markers in microglia and inflammatory activated microglia (mean ± SD; *n* = 3). **b** Cathepsin L; S; and B activity in the protein level (mean ± SD; *n* = 3). **c** mRNA level of inflammatory markers in inflammatory microglia treated Gel only; Drug only; and Drug & Gel (mean ± SD; *n* = 3). Statistical analysis was performed using a one-way analysis of variance (ANOVA) followed by Tukey’s *post hoc* test (*p < 0.1, **p < 0.01, and ***p < 0.001). **d** Protein level of EZH2 associated markers in inflammatory microglia. **e** Schematic illustration of EZH2 inhibition associated anti-inflammatory pathway signaling.

Epigenetic modulation of the retinal microenvironment has been studied as a promising therapeutic strategy in various retinal degenerative diseases^29,30^. For instance, as the EZH2 is known to contribute to rod photoreceptor death^8^ and microglial inflammation^9^, selective modulation of the EZH2 in a controlled manner could be a favorable approach for safe and effective therapeutic outcomes in retinal degenerative diseases which are associated with inflammation activities. Prior to conducting in vivo experiments, we performed in vitro experiments to confirm the anti-inflammatory effects of EZH2 inhibitor-loaded IRHs on inflammatory microglia. To test the anti-inflammatory effects of the EZH2-loaded IRH, we turned microglia into inflammatory microglia by applying TNF-α at 50 ng/mL to the media, and then various inflammatory activation markers were analyzed. Due the induced inflammation, the mRNA levels of TNF-α, IL1β, CCL2, and CCL5 were upregulated in the activated microglia (Fig. 5a; *see* Supplementary Fig. 1 for morphological changes of microglia). Consistent with the upregulated inflammatory markers, the protein activity levels of cathepsins L, S, and B increased (Fig. 5b). To test EZH2 inhibitor anti-inflammatory effects, we firstly treated EZH1/2 inhibitor (Valemetostat) and EZH2 inhibitor (Tazemetostat) to inflammatory microglia. Both EZH2 inhibitor drugs effectively inhibited the inflammatory response of microglia (Supplementary Fig. 2). Based on the result, we selected a Tazemetostat for selective inhibition of EZH2. Then, to examine transient anti-inflammatory effects of the IRH, the inflammatory microglia were incubated for 1 day at four different conditions: 1) with media only (Control), 2) with hydrogel only (Gel only), 3) EZH2 inhibitor only (Drug only), and 4) EZH2 inhibitor-loaded hydrogel (Drug & Gel) (*see* X-axis titles of bar graphs shown in Fig. 5c). As expected, both the Drug only and the Drug & Gel groups effectively reduced inflammatory responses in terms of the mRNA expression levels (red and green bars in Fig. 5c). It is worth to note that the EZH2 inhibitor loaded in the hydrogel (i.e., Drug & Gel group) did not lose drug activity and had anti-inflammatory effects similar to those of the EZH2 inhibitor-only treatment (i.e., Drug only group); statistical difference was not found in all but the CCL2 between the two groups (*compare* red and green bars in Fig. 5c) Therefore, EZH2 inhibitor delivery via IRH could be an effective treatment as an anti-inflammatory therapy for RP.

To further study the epigenetic modulatory effect of EZH2 inhibition on the induction of anti-inflammatory responses, the EZH2-associated signaling pathway was also investigated. In a previous study, IRF1/STAT1 signaling was suggested to be an EZH2-associated pathway that affects inflammatory mediators in microglia during neuroinflammation^9^. In our protein level analysis, the expression levels of p-STAT1, STAT1, and IRF1 were maintained similar in the Gel only group compared to the Control group (compare the first two columns of Fig. 5d), which means treating with hydrogel only did not affect epigenetic regulatory signaling. In contrast, Drug only and Drug & Gel groups downregulated p-STAT1 and IRF1 protein expression whereas the total protein of STAT1 was maintained, indicating successful inhibition of IRF1/STAT1 expression (Fig. 5d). These results show that EZH2 inhibitor effect is maintained even it loaded in the hydrogel. Consistent with the previous work, our results showed the EZH2 inhibitor-loaded hydrogel effectively attenuated the IRF1/STAT1 expression regulating inflammatory mediators in inflammatory microglia. Taken together, the EZH2 inhibitor-loaded IRH showed the potential for anti-inflammatory therapy in inflammatory microglia by reducing IRF1/STAT1 signaling, which regulates inflammation (Fig. 5e).

### Protecting photoreceptor death using the anti-inflammatory effect of on-demand EZH2 inhibition of inflammatory microglia

Prior to the intraocular injection, we tested the injectability of the hydrogel using both 31 G and 33 G Hamilton needles: The hydrogel was injectable at all crosslinker concentrations such as 0.25, 0.5, and 1 mM (Supplementary Fig. 3). As the IRH injection into the eyeball may elevate the intraocular pressure (IOP), we measured the IOPs of mouse eyes before and after the injection. First, 1 μL of the hydrogel fabricated with a 1 mM crosslinker was injected into *wt* and *rd10* mice (*see* Experimental Section); The IOP was analyzed both after 3 h of stabilization (i.e., day 0) and after 2 weeks (i.e., day 14) from the injection (Supplementary Fig. 4). Although our IOP measurements does not show transient changes, the measurements showed that the IRH injection did not alter the IOP after the recovery from the injection, remaining in the normal IOP range (10–20 mmHg) in both *wt* and *rd10* mice. This result confirms the fabricated IRHs are unlikely to cause any adverse event associated with the IOP such as glaucoma.

**Fig. 6:**
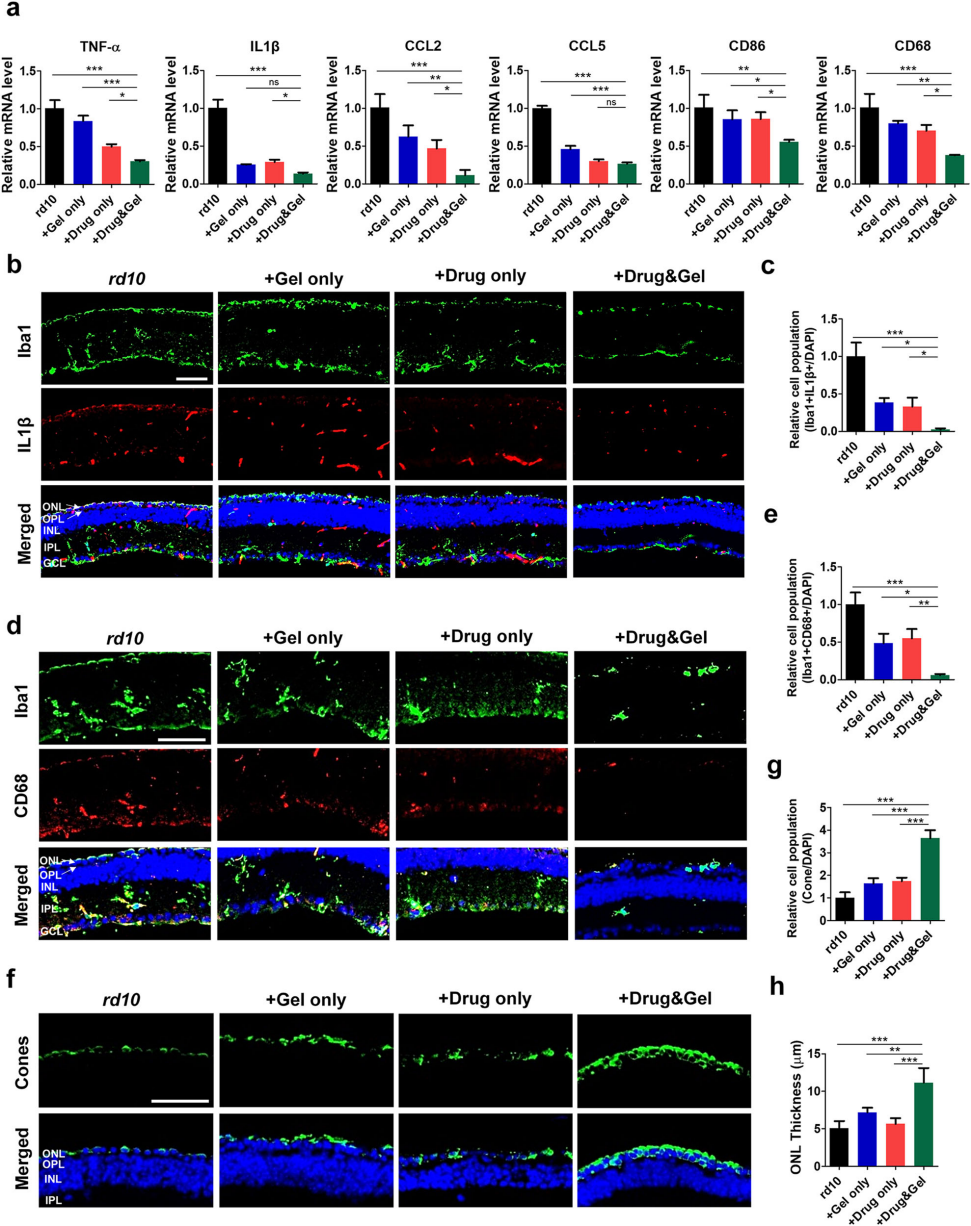
EZH2 inhibitor loaded-IFRep gel suppressed inflammatory microglia and protected photoreceptor in vivo. **a** mRNA level of inflammatory markers in *rd10* retina (mean ± SD; *n* = 3). **b** Immunofluorescence staining of microglia (Iba1) and inflammatory marker (IL1β) in *rd10* retina. ONL Outer Nuclear Layer (photoreceptor cell bodies), OPL Outer Plexiform Layer, INL Inner Nuclear Layer (bipolar cell bodies), IPL Inner Plexiform Layer, GCL Ganglion Cell Layer. **c** Quantification of inflammatory microglia (Iba1 + IL1β+) in immunofluorescence images (mean ± SD; *n* = 3). **d** Immunofluorescence staining of microglia (Iba1) and phagocytic marker (CD68) in *rd10* retina. **e** Quantification of inflammatory microglia (Iba1 + CD68+) in immunofluorescence images (mean ± SD; *n* = 3). **f** Immunofluorescence staining of survival cone cells in *rd10* retina. **g** Quantification of cone cells in immunofluorescence images (mean ± SD; *n* = 3). **h** Out nuclear layer (ONL) thickness after treatment (mean ± SD; *n* = 3). Statistical analysis was performed using a one-way analysis of variance (ANOVA) followed by Tukey’s *post hoc* test (*p < 0.1, **p < 0.01, and ***p < 0.001). Scale bar indicates 50 μm and applies to all images.

We investigated the therapeutic efficacy of EZH2 inhibitor-loaded IRHs in vivo with *rd10* mice, which is a widely-studied RP model. Hydrogel and/or drug were intravitreally injected to the 5-week-old (i.e., post-natal week 5 or PW5) *rd10* mice in three different groups: 1) hydrogels without any drug (Gel only group; *n* = 6), 2) EZH2 inhibitor in a form of solution without hydrogels (Drug only group; *n* = 6), and 3) EZH2 inhibitor-loaded hydrogels (Drug & Gel group; *n* = 6). After 2 weeks from the injection (i.e., PW7), retinal tissues were harvested and analyzed at genetic and protein levels to examine the degree of inflammation activities. First, the mRNA levels of six inflammatory markers (i.e., TNF-α, IL1β, CCL2, CCL5, CD86, and CD68) were plotted for the three groups which got intravitreal injections (blue, red, and green bars in Fig. 6) along with the control *rd10* mice (no injection; black bars in Fig. 6). Consistent with the previous result (Fig. 5c), all markers were downregulated in both the Drug only and the Drug & Gel groups compared to those of the *rd10* group, indicating a therapeutic effect of the EZH2 inhibitor in the in vivo model (*compare* black and red/green bars in Fig. 6a). However, it is important to note that the inflammatory response was even more significantly reduced when the EZH2 inhibitor and the IRH were delivered together (compare red and green bars in Fig. 6a), demonstrating the successful intraocular drug delivery capacity of the hydrogel. The on-demand release of the drug through the hydrogel can cause these results, which can retain the drug’s therapeutic effect in the eye. The Gel only treatment somehow also induced anti-inflammatory effects at the mRNA level (*compare* black and blue bars in Fig. 6a). This result indicates the anti-inflammatory effect of the HA hydrogel itself: a previous study reported, indeed, the high molecular-weight HA hydrogel (>1.0 × 10^6 ^Da) attenuated inflammatory receptors such as IL-1R1 and MyD88, reducing the inflammatory response^31,32^. Therefore, our HA-based IRHs might have boosted anti-inflammatory effects in addition to the EZH2 inhibitor due to the anti-inflammatory characteristic of the functional material, making the HA hydrogel even more attractive as on-demand drug delivery carriers.

To localize the inflammation activities in the retinal tissues, we additionally performed immunofluorescence staining. In comparison with the control group, the recruited microglia (Iba1) in the retina were lowest in the Drug & Gel group but they were quite decreased in both Gel only and the Drug only groups as well (first row of Fig. 6b). The reduced population of recruited microglia indicates reduced inflammatory response^33^. Moreover, the numbers of inflammatory microglia (stained by both Iba1 and IL1β) were effectively reduced in the Drug & Gel group (third row of Fig. 6b). The quantification of inflammatory microglia cell population clearly showed a substantial reduction in the Drug & Gel group (0.023 ± 0.018; All data in Fig. 6c, e, g were normalized by the control group of each figure) (Fig. 6c). It has been known that phagocytosis of microglia happens in inflammatory environments^4^. Thus, phagocytic microglia were also identified by expressing both Iba1 and CD68 in the retinas we tested (Fig. 6c), clearly displaying considerably reduced phagocytosis. The portion of the phagocytic microglia was most diminished when the EZH2 inhibitor was delivered with the IRH (0.061 ± 0.013) (Fig. 6e).

Lastly, to explore whether photoreceptors were saved by our treatments, we stained cone cells using immunofluorescence methods (Fig. 6f): as shown with a much thicker and brighter green signal band in the top right image, cone cells appeared to be most spared in the Drug & Gel group (3.64 ± 0.36). Further quantification counting of cone cells showed the Drug & Gel treatment significantly improved the cone cell survival by up to about four times compared to that of the control group (1.00 ± 0.25) and about two times compared to those of the Gel only (1.63 ± 0.24) and the Drug only (1.75 ± 0.15) treated groups (Fig. 6g). We also measured the thickness outer nuclear layer (ONL; shown as green in top row of Fig. 6f) as another indicator of degenerative progression of RP. The ONL thickness decreased to 5.05 ± 0.99 μm in the untreated *rd10* mouse group (black bar of Fig. 6h) but was maintained in the Drug & Gel group at 11.15 ± 1.96 μm (green bar of Fig. 6h), which is close to the initial ONL thickness at the time of treatment (~13.25 μm) (Supplementary Fig. 5). All of these results indicate that the Drug & Gel treatment effectively delayed the pathological progression of the retinal degenerative disease and saved cone cells. Collectively, on-demand EZH2 inhibitor delivery using the IRH may effectively alleviate vision loss caused by RD.

### Confirmation of delayed retinal degeneration through light-evoked response in the retina

**Fig. 7:**
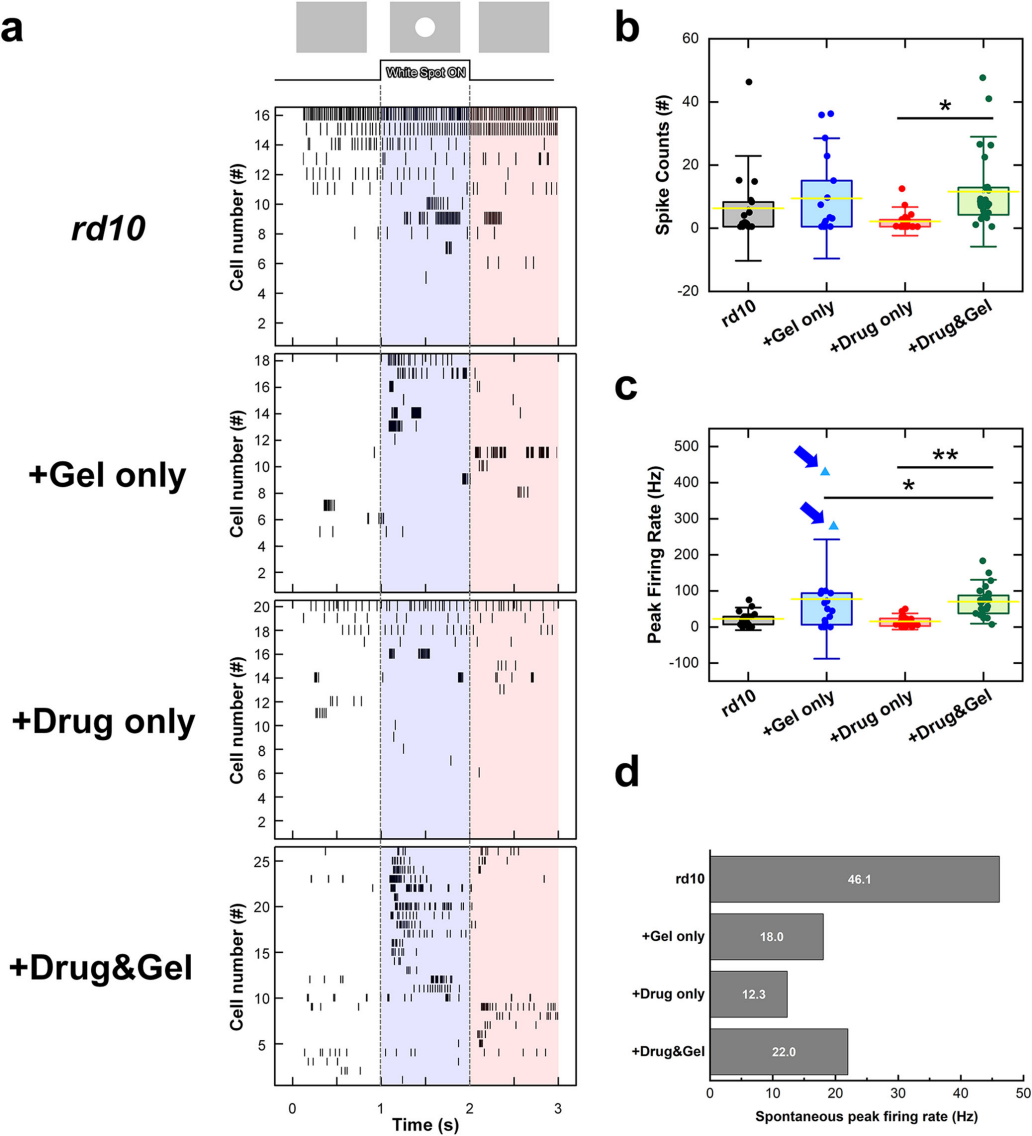
Light-evoked spiking activities from control; Gel only; Drug only; and Drug & Gel groups. **a** The raster plots of each group. The 1-sec-long white spot was projected onto the photoreceptor layer of target cells. Blue vertical band indicates the time range of spot flash was exposed; and the elicited spikes mean ON response. On the contrary; red vertical band indicates the OFF responses. Control (18 cells from 2 retinas); Gel only (18 cells from 2 retinas); and Drug only (20 cells from 4 retinas) groups showed spontaneous firing or silent response. However, Drug & Gel group (27 cells from 3 retinas) has relatively distinctive ON or OFF responses. **b** Spike counts; and **c** peak firing rate of a recorded RGC in each group. Two outlier cells of Gel group were marked with the blue arrows. Yellow Bars represent means and error bars indicate SDs. Statistical significance was assessed using the one-way ANOVA with Holm–Sidak *post hoc* comparisons (^∗^*p* < 0.05 and **p < 0.01). **d** Maximum value of spontaneous peak firing rate computed in every 20-ms-long bin.

In the retina, rod and cone photoreceptors transduce light signals into physiological signals, then their postsynaptic neurons such as bipolar, horizontal, and amacrine cells perform complex neural computations. At the final stage in the retina, retinal ganglion cells (RGCs) transmit visual information to the brain in a form of spike trains. Therefore, the degeneration/death of photoreceptor cells substantially alters spiking activities of RGCs. To investigate the potential delay in the loss of physiological visual function after our treatment using IRHs, the light-evoked responses were recorded from the RGCs of *rd10* mice non-treated and treated with Gel only, Drug only, and Drug & Gel (Fig. 7a). In response to stationary 1-sec-long white spot flashes, spiking responses were consistently elicited in RGCs of the Drug & Gel treated mice, and responses were strong enough to unambiguously classify most RGCs (24 cells out of 27 recorded cells) into either ON or OFF type (bottom raster plot of Fig. 7a). However, the *rd10* and Drug only treated groups showed spontaneous spiking activities throughout the entire recording period or a bunch of spikes temporally (first and third raster plots of Fig. 7a). These results are representative of the phenomena observed in degenerate retinas, which are known to be due to fluctuations in cell membrane potential of RGCs^34,35^. In contrast with the *rd10* and Drug only groups, it was possible to distinguish the RGC type (i.e., ON or OFF) in some cells of the Gel only group (second raster plot of Fig. 7a). However, there are RGCs that did not respond to the light stimulus at all, as seen in the *rd10* and the Drug only groups.

For more quantitative analyses, we also computed both spike count and peak firing rate (PFR) of the light responses (Fig. 7b, c; *see* Experimental Section). The RGCs of the Drug & Gel group generated 11.57 ± 11.59 spikes in average and showed the PFR of 70.06 ± 40.57 Hz in average. In contrast, the average spike counts of the *rd10*, Gel only, and Drug only groups (6.30 ± 11.07, 9.44 ± 12.70, and 2.17 ± 3.01 spikes, respectively) were relatively lower than those of the Drug & Gel treated mice (Fig. 7b). Although only one statistical significance was found in spike counts between Drug only and Drug & Gel groups (*p* < 0.05), due to the increased spontaneous spiking activities (Fig. 7d), PFR analyses clearly showed statistical significance in comparison with the Drug & Gel group. The average PFRs of the *rd10* (22.48 ± 20.90 Hz; *p* < 0.05) and Drug only (15.33 ± 14.96 Hz; *p* < 0.01) groups were significantly lower than that of the Drug & Gel group (70.06 ± 40.57 Hz). However, in the case of the Gel only group, owing to the two outlier RGCs (marked with blue arrows in Fig. 7c), the average PFR was higher than that of the other groups, and the standard deviation was also high (77.35 ± 110.18 Hz). Without those two outliers, the PFR was reduced to 42.83 ± 39.54 Hz. In the spiking activity analyses, it is particularly noteworthy that the Drug only group did not show the saved physiological functions of photoreceptors (compare red and green plots in Fig. 7b, c). This is in contrast with the similar levels of anti-inflammatory effects between the Drug only and the Drug & Gel groups, which were confirmed in the mRNA and immunofluorescence assays both in vivo and in vitro (Figs. 5 and 6). The enhanced capability protecting photoreceptor functions of the Drug & Gel treatment may be due to the slow drug release characteristics of the IRH (Fig. 4).

**Fig. 8:**
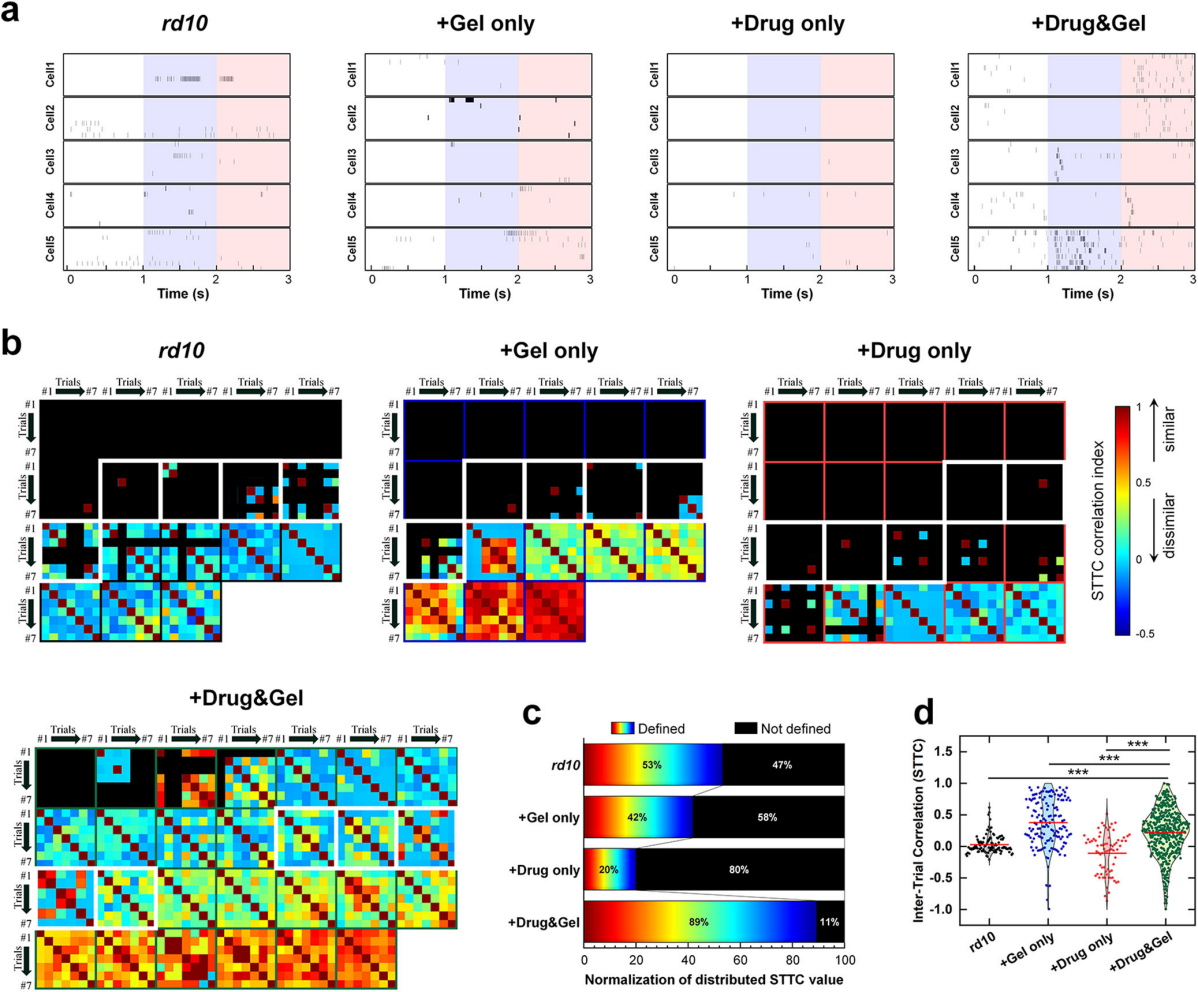
Spike timing consistency of light-evoked responses to 7 repeats of an identical light stimulus from *rd10*; Gel only; Drug only; and Drug & Gel groups. **a** Raster responses of a representative 5 RGCs from each group. **b** Color-coded heatmaps of the spike time tiling coefficients (STTCs) of light-evoked responses for each group. Each box with marked with color-solid line means STTC values in a given cell. Black color in matrices indicates no spiking activity was elicited in those trials. **c** Percentage of defined and not defined STTC value from each group. The number of defined/not defined STTC value was normalized according to the total number of RGCs and their repeats of light stimulation. **d** Violin plot showing distribution of STTCs from each group. Data points indicate STTC values of each pixel shown in STTC matrices in panel **b**. Red horizontal solid lines mean the average STTC values of each group. Statistical significance was assessed using the one-way ANOVA with Holm–Sidak *post hoc* comparisons (^∗∗∗^*p* < 0.001). All cells reported in Fig. 7 were also used in Fig. 8.

It has been known that multiple repeats of an identical stimulus evoke consistent spiking activities (i.e., similar spike trains arise across different repeats) in the healthy retinas while the consistency is reduced in the degenerate retinas^36^. To visualize the trial-to-trial consistency of the light responses we recorded from the five representative RGCs, we plotted the spiking activities evoked by seven different repeats of an identical light stimulus (Fig. 8a). The *rd10*, Gel only, and Drug only groups showed weak and inconsistent spiking patterns in response to the same light stimuli (Fig. 8a). We also calculated the spike time tiling coefficients (STTCs) (*see* equation (1) in Experimental Section) across those repeats in each RGC and then plotted the STTC matrices for all RGCs we measured (Fig. 8b). As shown in the first three subpanels of Fig. 8b, the black areas of the STTC heatmaps, which indicate lack of spiking activity, were larger in all but the Drug & Gel group. To quantify the portion of non-responsive RGCs in each group, we further calculated the percentages of defined and undefined STTC values, and then normalized them according to the number of recorded RGCs (Fig. 8c). Although STTCs were still computed in about half of RGCs of *rd10* mice (the first row Fig. 8c), it was primarily because the increased spontaneous activities (Fig. 7d): the spontaneous peak firing rates were 46.1, 18.0, 12.3, and 22.0 Hz in the *rd10*, Gel only, Drug only, and Drug & Gel groups, respectively. Particularly, the higher spontaneous activities resulted in near zero STTC in average (black plot of Fig. 8d). In sharp contrast, STTCs were well defined in most RGCs of the Drug & Gel group (STTC values were defined in 89% of all pairs of recordings as shown in the last row of Fig. 8c); the average STTC was also higher than that of the control group (0.22 ± 0.37 and 0.03 ± 0.14 for the Drug & Gel and *rd10* groups, respectively; Fig. 8d).

Once again, it is quite intriguing that the Drug only treatment demonstrated poor performance in the response consistency analyses, which was even worse than the outcome of the Gel only group (*compare* the middle two rows/columns in Fig. 8c, d). This trend is consistent with our response magnitude analyses (Fig. 7b, c). These results may have two implications: 1) one time injection of the EZH2 inhibitor may not be therapeutically effective, probably due to rapid posterior clearance^10^, and 2) the long-term implantation of HA-based hydrogel may protect the physiological function of photoreceptors, maybe by the reduced inflammation activities (Fig. 6). Actually, the average STTC of the Gel only group was even higher than that of the Drug & Gel group (0.38 ± 0.40 and 0.22 ± 0.37 for the Gel only and the Drug & Gel groups, respectively; Fig. 8d). However, this elevated result was due to the two outlier cells we mentioned in the PFR analysis (Fig. 7c). They showed red hot STTC matrices (the last two of second subpanel in Fig. 8b) and data points clustered near 1 in the violin plot (blue graph in Fig. 8d). Other than those two RGCs, most of RGCs in the Gel only group showed absence of light-evoked response, making it the second highest in terms of the percentage of undefined STTC values (i.e., 58% of all trials) (Fig. 8c).

In Fig. 8c, the percentage of defined STTC value in the control group (53%) was higher than those of the Gel only (42%) and Drug only (20%) groups. It was primarily because the spontaneous activities were relatively higher in the *rd10* and Drug & Gel groups (Fig. 7d). However, as aforementioned, the calculated average STTC of the *rd10* group was low owing to inconsistent responses. Notably, although several RGCs of the Drug & Gel group also showed spontaneous activities during the pre-stimulus period (Fig. 7a), the light-evoked responses were relatively strong and consistent after the light stimulus.

Collectively, our results indicate that treatment using an IRH loaded with an EZH2 inhibitor can effectively slow down the degradation of physiological functions of the retina, in addition to the reduced inflammation activities, both which arise from RP. Similar to our study, there were several endeavors to preserve morphology and function of the retina during RD with diverse methods such as electric stimulation^37,38^, repeating intravitreal drug injection^4,7,39^, or genome editing^40,41^. However, these methods have exhibited some drawbacks, including frequent application of electric pulses^37,38^ and repeating injection^39^ as well as low-transfection efficiency of virus vectors. The uniqueness of our study, compared to those existing literature, is that the treatment effect was maintained for a long period by loading the drug in IRH as well as the protected physiological retinal functions were examined at the level of single spike in each RGC using a patch-clamp recording technique. However, it seems unclear whether the treatment can preserve the retinal circuit comprehensively involved in forming visual percepts. Furthermore, another limitation to be considered is that we started the treatment at PW5 but there is a possibility that initiating treatment at an early degeneration stage (e.g., *rd10* mice at PW3) might have led to a more favorable outcome. Unfortunately, however, due to the small size of the eyeball, the injection of IRH was not feasible earlier than 3 weeks, which would not be the case in clinical cases because human eyeballs are much bigger than mouse ones. In the future, it appears necessary to enhance the anti-inflammatory effect to preserve the retinal circuit more intact for sophisticated neural computations by optimizing the concentration of the drug, injection timing, and so on. Moreover, the inflammation-responsive hydrogel can respond to disease states, but enabling long-term drug release for clinical application is necessary. The advanced inflammation-responsive hydrogel can be developed via the optimization of cathepsin-cleavable crosslinker concentration and hydrogel mechanical properties in future studies. Although it may be almost impossible to reverse RD using our approach, slowing/stopping degeneration of the retina would be beneficial for microelectronic retinal prosthetic technologies because our previous studies using RGCs of the healthy retinas demonstrated favorable response characteristics to electric stimulation, such as naturalness of spiking activities and increased ON/OFF cell type specificities, for high-quality artificial vision^42^.

## Discussion

In this study, HA-based inflammation-responsive hydrogel for on-demand anti-inflammatory therapy for a retinal degenerative disease was developed. The inflammation-responsive hydrogel revealed drug release in a controlled manner according to various concentrations of cathepsins and inflammatory conditions. Our in vitro experiments demonstrated that the hydrogel was suitable for intraocular injection because of properties such as injectability, biocompatibility, and transparency. Also, an on-demand anti-inflammatory effect on inflammatory microglia was achieved via epigenetic modulation of EZH2 inhibitor released from the hydrogel. The intravitreal injection of the EZH2 inhibitor-loaded hydrogel for outer retinal degenerative model *rd10* mice induced an anti-inflammatory effect in the retina environment and protected cone cell death of photoreceptors. In particular, ganglion cell response results showed delayed retinal degeneration and vision loss in the *rd10* mice model. Collectively, HA-based inflammation-responsive hydrogel exhibited efficient drug delivery according to the disease activity for anti-inflammatory therapy. This therapeutic strategy suggested effective treatment potential in progressive retinal degenerative disease. The hydrogel platform developed in this study can be applied to diverse inflammatory diseases that require minimally invasive treatment in combination with various drugs. For clinical applications, further study is necessary to develop inflammation-responsive hydrogels that actively control disease and enable long-term drug release.

## Methods

### Fabrication of Dibenzocyclooctyne-conjugated hyaluronic acid (DBCO-HA)

Hyaluronic acid (HA, 2 M Da; SK Bioland, Seoul, South Korea) was dissolved in DMSO:distilled water (1:1) solution at a concentration of 0.5 *wt*%. Then, 0.6 mM 1-ethyl-3-(3-dimethylaminopropyl) carbodiimide hydrochloride (EDC) was dissolved in the HA solution, followed by 0.6 mM N-hydroxysuccinimide (NHS) was dissolved in the solution for 30 min. To the prepared solution, 0.4 mM of DBCO-PEG4-amine (#A103P, Click Chemistry Tools, Scottsdale, USA) was slowly added; the reaction was kept at room temperature for 24 hours. After the reaction was finished, the solution was transferred to a 25 kDa molecular weight cut-off (MWCO) dialysis tube and dialyzed in deionized water (DI) for 3 days. The dialysate was changed every 10 h. The DBCO-conjugated HA (DBCO-HA) solution was sterilized by a 0.22 µm syringe filter and freeze-dried for 3 days. The successful conjugation of DBCO to HA was confirmed via proton nuclear magnetic resonance (^1^H-NMR) spectroscopy (the sample concentration of the DBCO-HA solution was prepared at 2 wt% in deuterium oxide).

### Preparation of cathepsins-cleavable crosslinked DBCO-HA hydrogel

Cathepsins-cleavable peptide crosslinker, azidoacetyl-ARLRK-azidoacetyl (MW: 808.51 Da, Purity: 99%; model number, Peptron, Daejeon, South Korea), was designed based on our previous publication^23^. After 2 wt% DBCO-HA was dissolved in DI water, the cathepsins-cleavable crosslinker was added at various concentrations: 0.25 mM, 0.5 mM, and 1 mM. The mixture of solutions was incubated at 37 °C for 30 min.

### Mechanical property measurement

The rheology of the fabricated hydrogel was analyzed using rheometer (Anton Paar, Graz, Austria). The measurements were performed at oscillating frequencies ranging from 0.1 to 100 rad/sec at 0.1% strain at room temperature. Both storage modulus (G’) and loss modulus (G”) were calculated with the software of the rheometer. Also, the stiffness of the hydrogel was analyzed with Instron (Instron 5900, Instron Corporation, Norwood, Massachusetts, USA). By compressing the hydrogel, the stress-strain curve was measured, and then Young’s modulus was obtained as the slope of the linear region (first 10% of strain) in that stress-strain curve.

Scanning electron microscope *(SEM) imaging*: The freeze-dried hydrogels were dumped into liquid nitrogen to get clear cross-sections of the samples during cutting process. The frozen samples were cut with a blade. Then, the cut samples were placed on a carbon tape and the surface was coated with platinum before loading into a SEM (Teneo VS™, Thermo Fisher Scientific, Waltham, Massachusetts, USA), which imaged the prepared samples.

### Characterization of drug release profile from inflammation-responsive hydrogel

Dextran (MW: 4 kDa) was loaded in the hydrogel to quantify dye release rate at various concentrations of cathepsins. Dextran-loaded hydrogels were placed in three 24 transwell inserts and incubated in the control media (DMEM/F-12 media), conditioned media of microglia, and activated microglia in DMEM/F-12 media, which were all maintained at 37 °C. The conditioned media of microglia was prepared by culturing microglia in DMEM/F-12 for 1 day. A conditioned medium for activated microglia was prepared by inducing inflammatory microglia with 50 ng/ml of TNF-α (#210-TA, R&D System) in DMEM/F-12 for 1 day. The conditioned media of activated microglia was harvested of supernatant of culturing media in DMEM/F-12 for 1 day. The inserts were transferred to a new 24 well plate, and the fluorescence intensity was measured using an in vivo imaging system (IVIS, Caliper Life Sciences, Waltham, Massachusetts, USA) at the time point of 0, 1, 3, and 5 days. In addition, the hydrogels loaded with EZH2 inhibitors, Tazemetostat (EPZ-6438, Selleckchem, Houston, Texas, USA), were cultured in a control media (DMEM/F-12 media), conditioned media of microglia and activated microglia in DMEM/F-12 media, which were all maintained at 37 °C. The supernatants were collected at the time point of 0.125, 0.25, 0.5, 1, 3, and 5 days and quantified via absorbance rate of ultraviolet (UV) at 256 nm. Then, we investigated EZH2 inhibitor release profiles in responses to different concentrations (i.e., 10 and 100 ng/ml) of cathepsins (L, S, and B subtypes were all mixed) added in phosphate buffered solution (PBS) at 37 °C. Up to five days, the supernatants were collected each day for UV absorbance measurement. Cathepsins were added at every time point of 0.125, 0.25, 0.5, 1, and 3 days.

### Cell culture for in vitro experiments

Human microglia cell line SV40 was purchased from Applied Biological Material Inc. (T0251, Milton, Canada) and cultured in Dulbecco’s Modified Eagle Medium/Nutrient Mixture F-12 (DMEM/F-12 in 1:1) (#11320-033, ThermoFisher, Waltham, Massachusetts, USA) supplemented with 10% fetal bovine serum (FBS) and 1% penicillin/streptomycin (PS). For the SV40 cell culture, collagen solution (50 μg/mL in 20 mM acetic acid) was coated on the culture plate and then washed with PBS 3 times before the culture of SV40 cells. Mouse fibroblast (NIH3T3) was obtained from American Type Culture Collection (NIH/3T3 - CRL-1658, Manassas, VA, USA), and cultured in DMEM (#11995-065, ThermoFisher, Waltham, Massachusetts, USA) supplemented with 10% FBS and 1% PS. The cells were incubated in the humid atmosphere with 5% CO_2_ at 37 °C.

### Hydrogel in vitro cytotoxicity testing

The in vitro cytotoxicity of the fabricated hydrogel was investigated using Cell Counting Kit-8 assay (CCK-8, Dojindo, Kumamoto, Japan). NIH3T3 cells were seeded in 24 well plates and cultured with the hydrogels placed in the 24 transwell insert. Fibroblasts (NIH3T3) are representative cell source to test biomaterial cytotoxicity because these cells have high growth rates and biological responses^43,44^. Also, it has been reported that retinal pigment epithelium-like cells and retinal ganglion-like cells can be derived from fibroblasts^45,46^. Therefore, we performed our cell cytotoxicity test of the hydrogel using NIH3T3 cells. After incubation with the hydrogels, cells were washed with PBS and immersed in DMEM-free media with 10% CCK-8 solution for 1.5 h at 37 °C. The supernatant was collected and measured by absorbance of light at 450 nm in wavelength using a microplate reader. Staining of live and dead cells was performed with a Live & Dead/Cytotoxicity Assay Kit (#L3224, Invitrogen, Waltham, Massachusetts, USA) according to the manufacturer’s protocol. A mixture of 4 mM calcein AM (green) and 2 mM ethidium homodimer-1 (red) was added to the culture media and incubated for 30 min at 37 °C. The stained cells were visualized with a confocal microscope (Carl Zeiss, Jena, Germany).

### In vitro study of anti-inflammatory effects on microglia

SV40 cells were cultured with 50 ng/ml of TNF-α (#210-TA, R&D systems, Minneapolis, Minnesota, USA) for 1 day to induce inflammatory microglia. For the study of anti-inflammatory effects, inflammation-activated microglia were treated with three different conditions: hydrogel only (Gel only), 3) EZH2 inhibitor only (Drug only), and 4) EZH2 inhibitor-loaded hydrogel (Drug & Gel).

### Cathepsin activity assay

For confirmation of secreted cathepsins from non-activated and activated microglia, the conditioned media was harvested after 1 day of incubation of microglia in the DMEM/F-12 media. The *rd10* model retina tissues were used for cathepsin activity assay in vivo. To analyze the activities of cathepsin L, S, and B, fluorescence-based assay kits (#K142, #K144, #K140, Biovision, Milpitas, California, USA) were used. The procedure was performed according to the manufacturer’s instructions. The fluorescence intensity of samples was measured with a fluorescence plate reader at 400/505 nm for excitation/emission wavelengths, respectively.

### Quantitative real-time polymerase chain reaction (real-time PCR) for mRNA assay

The gene expression levels of cathepsins (cathepsin L, S, and B) and inflammatory markers (i.e., TNF-α, IL1β, CCL2, CCL5, CD86, and CD68) were analyzed by real-time PCR assay. We isolated mRNA from microglia and retina tissue using a RNeasy Mini Kit (#74104, QIAGEN, Hilden, Nordrhein-Westfalen, Germany), and synthesized to cDNA using a SuperScript IV VILO Master Mix (#11755050, Invitrogen, Waltham, Massachusetts, USA). Gene expression level was performed with a SYBR Premix Ex Taq (#RR420A, Takara, Kusatsu, Japan) according to the manufacturer’s instruction. The sequences of all primers used in this study are presented in Table 1. The expressed gene levels were normalized to GAPDH which served as a house-keeping gene, and the relative gene levels were calculated by the 2^−△△CT^ method.

**Table 1:** List of primer sequences used in real-time PCR experiments in the present study.

Gene	Forward (5” → 3”)	Reverse (3” → 5”)
hGAPDH	CACCATTGGCAATGAGCGGTTC	AGGTCTTTGCGGATGTCCACGT
hTNF-α	GTGCCTATGTCTCAGCCTCTTC	GCCATAGAACTGATGAGAGGGA
hIL1β	CCACAGACCTTCCAGGAGAATG	GTGCAGTTCAGTGATCGTACAGG
hCCL2	AGAATCACCAGCAGCAAGTGTCC	TCCTGAACCCACTTCTGCTTGG
hCCL5	CCTGCTGCTTTGCCTACATTGC	ACACACTTGGCGGTTCTTTCGG
hCD86	CCATCAGCTTGTCTGTTTCATTCC	GCTGTAATCCAAGGAATGTGGTC
hCD68	CGAGCATCATTCTTTCACCAGCT	ATGAGAGGCAGCAAGATGGACC
hCathepsin L	GAAAGGCTACGTGACTCCTGTG	GTCTACCAGATTCTGCTCACTC
hCathepsin S	TGGATCACCACTGGCATCTCTG	GCTCCAGGTTGTGAAGCATCAC
hCathepsin B	GCTTCGATGCACGGGAACAATG	CATTGGTGTGGATGCAGATCCG
mGAPDH	CATCACTGCCACCCAGAAGACTG	ATGCCAGTGAGCTTCCCGTTCAG
mTNF-α	GGTGCCTATGTCTCAGCCTCTT	GCCATAGAACTGATGAGAGGGAG
mIL1β	TGGACCTTCCAGGATGAGGACA	GTTCATCTCGGAGCCTGTAGTG
mCCL2	GCTACAAGAGGATCACCAGCAG	GTCTGGACCCATTCCTTCTTGG
mCCL5	CCTGCTGCTTTGCCTACCTCTC	ACACACTTGGCGGTTCCTTCGA
mCD86	ACGTATTGGAAGGAGATTACAGCT	TCTGTCAGCGTTACTATCCCGC
mCD68	GGCGGTGGAATACAATGTGTCC	AGCAGGTCAAGGTGAACAGCTG
mCathepsin L	GGAAAATGGAGGTCTGGACTCG	GTGTCATTAGCCACAGCGAACTC
mCathepsin S	GCATAGAGGCAGACGCTTCCTA	CCACTGCTTCTTTCAGGGCATC
mCathepsin B	AGTCAACGTGGAGGTGTCTGCT	GTAGACTCCACCTGAAACCAGG

### Immunofluorescent staining

For immunofluorescence staining of in vivo, retina tissues were fixed with 4% paraformaldehyde for 15 min, and embedded in optimal cutting temperature (OCT) compound for freezing. Tissues cryo-sectioned in 7 μm were stained with 1:250 diluted Anti-Iba1 antibody (#019-19741, Wako, Osaka, Japan), 1:200 diluted Anti-IL1β antibody (#sc-12742, Santa Cruz, Dallas, Texas, USA), 1:500 diluted Anti-TNF-α antibody (#ab183218, Abcam, Cambridge, Cambridgeshire, UK), 1:100 diluted Anti-CD68 antibody (#MCA1957T, Bio-Rad, Hercules, Contra Costa County, CA, USA), 1:500 diluted Anti-Cone arrestin antibody (#AB15282, Sigma-Aldrich, St.Louis, Missouri, USA), 1:100 diluted Anti-Cathepsin L antibody (#ab133641, Abcam, Cambridge, Cambridgeshire, UK), 1:300 diluted Anti-Cathepsin S antibody (#sc-271619, Santa Cruz, Dallas, Texas, USA), and 1:250 diluted Anti-Cathepsin B antibody (#ab214428, Abcam, Cambridge, Cambridgeshire, UK) as primary antibodies. The secondary antibodies were 1:500 diluted Donkey anti-Rabbit IgG antibody (#A21207, Invitrogen, Waltham, Massachusetts, USA) and 1:500 diluted Goat anti-Mouse IgG antibody (#A11001, Invitrogen, Waltham, Massachusetts, USA). The quantification of inflammatory microglia and cone cells was measured using immunofluorescence-stained retina cryosection. The inflammatory microglia were counted in the yellow area, overlapping green (Iba1) and red (IL1β or CD68) in the retina section using ImageJ. The cone cells were counted in cone arrestin antibody-stained green area using ImageJ. The measured area was normalized by calculating the DAPI (blue) area of the retina. The thickness of photoreceptor layer was averaged from randomly selected areas in three retinas to determine the precise effect of EZH2 inhibitor-loaded hydrogels.

### Western blot

For immunoblotting, prepared cell samples were lysed wih RIPA lysis buffer (#R0278, Sigma-Aldrich, St. Louis, MO, USA) including protease and phosphatase inhibitor cocktail (#ab201119, Abcam, Cambridege, Cambridgeshire, UK). Equal amounts of protein were loaded onto 4–15% gradient gel (Bio-Rad Laboratories, CA, USA) and transferred to polyvinylidene difluoride membranes (PVDF) (Bio-Rad Laboratories, CA, USA) followed by blocking with 5% BSA in TBST (TBS with 0.1% Tween 20) for 1 h in the room temperature. The membranes were incubated with primary antibodies including 1:5000 diluted anti-GAPDH antibody (#ab8245, Abcam, Cambridege, Cambridgeshire, UK), 1:1000 diluted anti-Phospho-STAT (#9167, Cell Signaling, Danvers, MA, USA), 1:1000 diluted anti-STAT1 antibody (#9172, Cell Signaling), and 1:1000 diluted anti-IRF1 antibody (#8478, Cell Signaling) in 5% BSA in TBST at 4°C overnight. Membranes were incubated with 1:1000 diluted HRP-conjugated anti-mouse or anti-rabbit IgG (#7074, #7076, Cell Signaling) at room temperature for 1 h. The immunoblots were visualized with the chmiluminescence system (#34095, ThermoFisher, Waltham, Massachusetts, USA), and images were taken using an iBright CL1500 imaging system (ThermoFisher, Waltham, Massachusetts, USA). The unedited images of immunoblotting are presented in the Supplementary Fig. 6.

### Animal preparation

The animal study was approved by Korea Institute of Science and Technology (KIST-5088-2022-05-077). All animal experiments were performed according to the institutional guidelines for the care and use of laboratory animals. Wild-type mice (C57BL/6J strain) were purchased from Daehan BioLink (Eumseong, South Korea). Retinal degeneration 10 (*rd10*) mice were used as a retinitis pigmentosa (RP) model. We purchased breeding pairs of *rd10* mice (B6.CXB1-*Pde6b*^rd10^/J) from the Jackson Laboratory (Bar Harbor, ME) and a colony had been maintained in the KIST animal facility. The *rd10* mouse is the most widely used animal model to study the model of autosomal recessive RP^47^. Photoreceptor degeneration in the *rd10* mice is known to well mimic typical pattern of human RP because it does not overlap with retinal development and allows light responses to be recorded for about a month after birth^48^. However, the *rd10* mice only can represent one RP genotype which carries a missense mutation in the *Pde6b* gene (phosphodiesterase 6B, cGMP, rod receptor, beta polypeptide)^47^. Therefore, to cover various RP genotypes such as *Crb1*^49^, other *rd* mice are needed.

### Intravitreal injection in vivo and retinal tissue preparation

To compare anti-inflammatory effectiveness of materials, we prepared 4 different mouse groups: 1) *rd10* control without any injection, 2) hydrogel only (Gel only), 3) EZH2 inhibitor only (Drug only), and 4) EZH2 inhibitor-loaded hydrogel (Drug & Gel). During the intravitreal injection, mice were kept anesthetized by isoflurane in oxygen. To inject prepared materials, we made a small hole which was punctured at slightly posterior to the limbus with a 30 G 1/2 needle. Subsequently, the prepared hydrogel, EZH2 inhibitor (Tazemetostat) of 1-2 μL was intravitreally injected into 5-week-old mice with a 33 G Hamilton needle (#NANOFIL, World Precision Instruments, Sarasota, Florida, USA). After 2 weeks from the injection, mice were anesthetized via inhalation of vaporizing isoflurane and sacrificed by cervical dislocation. Subsequently, the retina tissues were separated from the eyeball with fine forceps and then used for in vivo experiments.

### Patch-clamp recording & light stimuli

Physiological functions of retina tissues were examined at the level of single spike in each individual RGC using patch-clamping recordings. The separated retinas were mounted ganglion cell side up on a filter paper which had a small hole (~2 mm in diameter) at the center for allowing light stimuli. The prepared samples were placed on a slide glass and were constantly perfused at 4 mL/min with oxygenated Ames’ medium (Sigma Aldrich, St. Louis, MO, USA) which was maintained at 34–36 °C. The spiking activities of RGCs were recorded with a glass-pipette (8–11 MΩ) by using the cell-attached patch-clamping method. After removing the inner-limiting membrane, alpha RGCs with large somata (>20 μm) were targeted^50,51^. As ground electrodes, two silver chloride-coated silver wires shaped in balls were placed at the opposite sides of a recording chamber. Data were recorded and low-pass filtered at 2 kHz using an amplifier (MultiClamp 700B, Molecular Devices, Sunnyvale, CA).

For light stimuli, stationary 1-sec-long white spot flashes on a gray background were focused at the photoreceptor layer and were delivered at various diameters ranging from 100 to 1000 μm. All visual stimuli were repeated 5–7 times. The number of recorded RGCs was 16, 21, 20, and 27 from the control, Gel only, Drug only, and Drug & Gel groups, respectively.

### Analysis of light-evoked spiking responses

Patch-clamp recordings allowed us to perform sophisticated spike-level analyses of retinal neurons protected from degeneration. First, the timing of elicited spikes was detected from raw recordings by custom scripts written in MATLAB (MathWorks, Natick, MA, USA). Based on the light-evoked responses, the RGCs were classified into ON, OFF, ON-OFF, and unknown (contain no response cell or remaining cell except classified cell) subtypes. RGCs were not tested with moving stimuli for direction-selective subtypes^52–54^. The spikes of ON and OFF RGCs were counted for 1-sec-long presentation of light stimulus (*see* red band in Fig. 7a) and for 1 sec from the offset of the light (*see* blue band in Fig. 7a), respectively. However, in case of the unknown and ON-OFF type of RGCs, because it was difficult to determine the response polarity (i.e., ON or OFF), we averaged the spike counts from both ON and OFF response periods (for 2 s; both red and blue bands in Fig. 7a). Among the numbers of spikes arising from diverse spot sizes (i.e., from 100 to 1000 μm), we selected the biggest number of spikes for following analyses. Firing rates were computed using a bin size of 20 ms which was moved in 5 ms step; peak firing rate (PFR) was the maximum value of those computed firing rates. Also, we examined spike timing consistency by computing spike time tiling coefficient (STTC) across repeats^55^ using the following equation (1): STTC = 1/2 × [(*P*_*A*_ − *T*_*B*_)/(1 − *P*_*A*_*T*_*B*_) + (*P*_*B*_ − *T*_*A*_)/(1 − *P*_*B*_*T*_*A*_)]. *P*_*A*_ is the proportion of spikes from A which lie within ±time window (±Δt) of any spike from B (*P*_*B*_ calculated similarly), *T*_*A*_ is the proportion of total recording time which lies within ±Δt of any spike from A (*T*_*B*_ calculated similarly). We used Δt of 10 ms in this work.

The calculated STTCs were plotted as color-coded heatmaps (Fig. 8b). Each heat matrix of STTCs computed from a given cell is surrounded with thin color borders indicating different groups (i.e., black, blue, red, and green for control, Gel only, Drug only, and Drug & Gel, respectively). We used black color to represent the case where the STTC value was not defined due to the absence of spiking activity. The heat matrices were arranged in the following order: 1) low to high number of defined STTC values and 2) low to high average STTC value. We selected 5 RGCs in the middle of the arranged heat matrices (*see* thick white borders surrounding them) and their raster plots were shown as representative cells (Fig. 8a). The percentage of ‘*undefined*’ and ‘*defined*’ STTC values in each group was calculated (Fig. 8c). The defined STTC values were also presented as data points in violin plots (Fig. 8d). Spontaneous activities were evaluated by calculating pre-stimulus period of 500 ms (Fig. 7d).

### Statistical analysis

All data were expressed as mean ± standard deviation. Data shown in Figs. 1, 4, 5, and 6 were analyzed using a one-way analysis of variance (ANOVA) followed by Tukey’s *post hoc* test in Prism. Data shown in Figs. 7 and 8 were analyzed using a one-way analysis of variance (ANOVA) followed with a Holm–Sidak *post hoc* test in Origin. The statistical significance was shown with *p < 0.05, **p < 0.01, ***p < 0.001 and ****p < 0.0001.

### Reporting summary

Further information on research design is available in the Nature Research Reporting Summary linked to this article.

### Supplementary information

Supplemental Material

Reporting Summary

## Data Availability

All data generated or analyzed during this study are included in this published article and its supplementary information files. Those datasets used and/or analyzed during the current study available from the corresponding author on reasonable request.

## Code availability

The underlying code including MATLAB codes utilized to analyze spiking activities of ganglion cells is not publicly available but may be made available to qualified researchers on reasonable request from the corresponding author.
