# Supplementary material for: Effective protection of photoreceptors using an inflammation-responsive hydrogel to attenuate outer retinal degeneration
Source: NPJ Regen Med. 2023 Dec 14;8:68. doi: 10.1038/s41536-023-00342-y (PMC10721838; doi:10.1038/s41536-023-00342-y)
Supplement: Supplementary file 1 — Supplemental Material [file 41536_2023_342_MOESM1_ESM.pdf]

Supplementary Information

Effective Protection of Photoreceptors Using an Inflammation-Responsive Hydrogel to Attenuate Outer Retinal Degeneration

Hyerim Kim[†]*, Hyeonhee Roh*[†]*, Sang-Heon Kim, Kangwon Lee*[*]*, Maesoon Im*[*]*, Seung Ja Oh*[*]

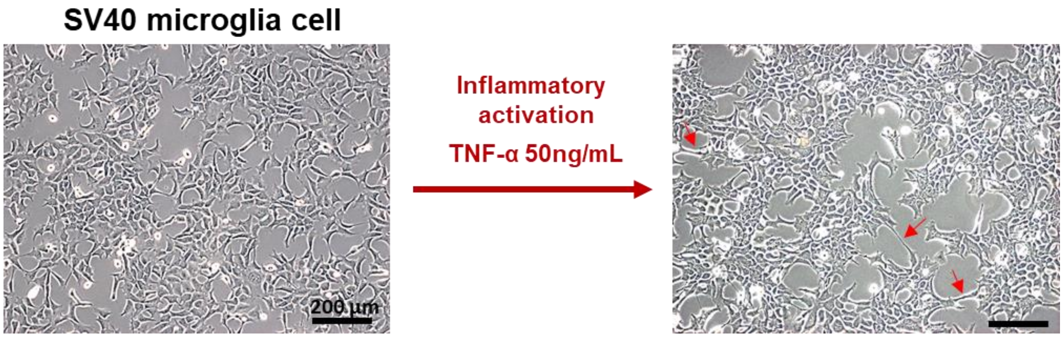

Supplementary Figure 1. Optical images of microglia cells (SV40) showed extended cell phenotype

The morphology of microglia cells was changed after inflammatory activation with 50 ng/mL TNF-α treatment. Scale bar indicates 200 μm and applies to all images.

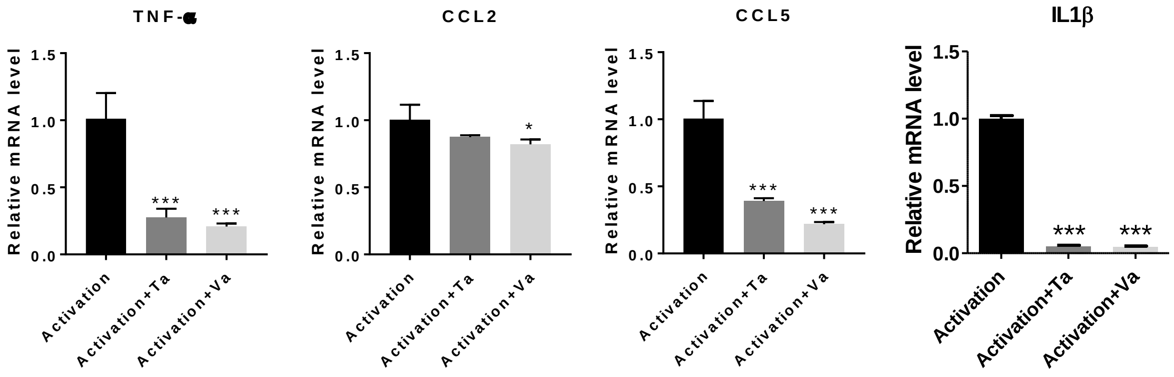

Supplementary Figure 2. The mRNA levels of inflammatory markers in inflammatory microglia. Inflammatory microglia were treated with Ta (Tazemetostat, EZH2 inhibitor) and Va (Valemetostat, EZH1/2 inhibitor) for 1 day (mean ± SD).

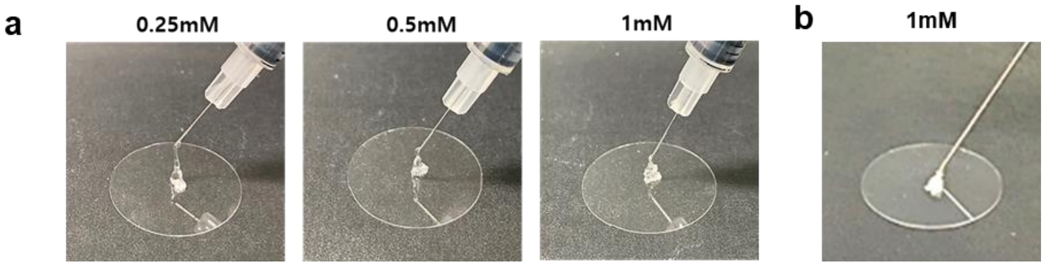

Supplementary Figure 3. Dispensing of HA-based inflammation-responsive hydrogels. a) A 31G Hamilton needle dispensed all hydrogels made with various cathepsins-cleavable crosslinker concentrations (0.25, 0.5, and 1 mM, respectively). b) Same as *a* but for a 33G needle. Hydrogel fabricated with 1 mM crosslinker was used.

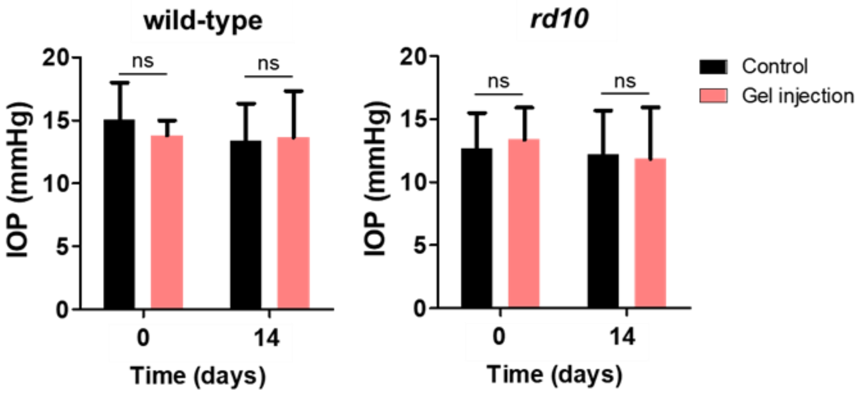

Supplementary Figure 4. Intraocular pressure (IOP) of wild-type and *rd10* mice eyes after hydrogel injection. After intravitreal injection of the hydrogel in mouse eye balls, IOPs were measured using iCare Pro (TA03) (mean ± SD).

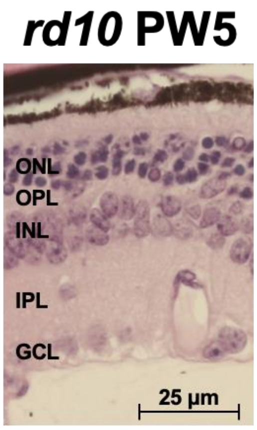

Supplementary Figure 5. An example of H&E staining of the *rd10* mouse retina at PW5. ONL: Outer Nuclear Layer (photoreceptor cell bodies); OPL: Outer Plexiform Layer; INL: Inner Nuclear Layer (bipolar cell bodies); IPL: Inner Plexiform Layer; GCL: Ganglion Cell Layer. Scale bar indicates 25 μm.

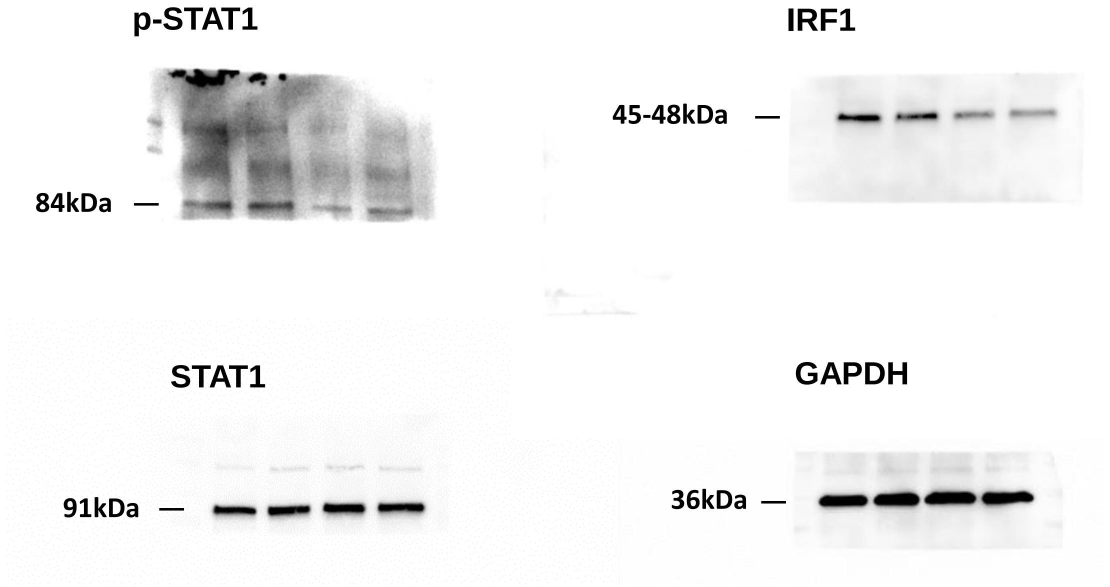

Supplementary Figure 6. Unedited images of immunoblotting presented in Figure 5d.
