# Supplementary material for: Effective protection of photoreceptors using an inflammation-responsive hydrogel to attenuate outer retinal degeneration
Source: NPJ Regen Med. 2023 Dec 14;8:68. doi: 10.1038/s41536-023-00342-y (PMC10721838; doi:10.1038/s41536-023-00342-y)
Supplement: Supplementary file 2 — Reporting Summary [file 41536_2023_342_MOESM2_ESM.pdf]

nature portfolio

Corresponding author(s): Kangwon Lee, Maesoon Im, Seung Ja Oh

Last updated by author(s): Aug 21, 2023

Reporting Summary

Nature Portfolio wishes to improve the reproducibility of the work that we publish. This form provides structure for consistency and transparency in reporting. For further information on Nature Portfolio policies, see our Editorial Policies and the Editorial Policy Checklist.

Statistics

For all statistical analyses, confirm that the following items are present in the figure legend, table legend, main text, or Methods section.

n/a	Confirmed	
☐	☒	The exact sample size (*n*) for each experimental group/condition, given as a discrete number and unit of measurement
☐	☒	A statement on whether measurements were taken from distinct samples or whether the same sample was measured repeatedly
☐	☒	The statistical test(s) used AND whether they are one- or two-sided<br>*Only common tests should be described solely by name; describe more complex techniques in the Methods section.*
☐	☒	A description of all covariates tested
☒	☐	A description of any assumptions or corrections, such as tests of normality and adjustment for multiple comparisons
☐	☒	A full description of the statistical parameters including central tendency (e.g. means) or other basic estimates (e.g. regression coefficient) AND variation (e.g. standard deviation) or associated estimates of uncertainty (e.g. confidence intervals)
☐	☒	For null hypothesis testing, the test statistic (e.g. F, t, r) with confidence intervals, effect sizes, degrees of freedom and P value noted<br>*Give P values as exact values whenever suitable.*
☒	☐	For Bayesian analysis, information on the choice of priors and Markov chain Monte Carlo settings
☒	☐	For hierarchical and complex designs, identification of the appropriate level for tests and full reporting of outcomes
☒	☐	Estimates of effect sizes (e.g. Cohen's *d*, Pearson's *r*), indicating how they were calculated

Our web collection on statistics for biologists contains articles on many of the points above.

Software and code

Policy information about availability of computer code

Data collection	For patch-clamp recordings, the timing of elicited spikes was detected from raw recordings by custom scripts written in MATLAB (MathWorks, Natick, MA, USA). The rheology of the fabricated hydrogel was analyzed using rheometer (Anton Paar, Graz, Austria). Both storage modulus (G′) and loss modulus (G″) were calculated with the software of the rheometer.
Data analysis	For patch-clamp recordings, the timing of elicited spikes was detected from raw recordings by custom scripts written in MATLAB (MathWorks, Natick, MA, USA).

For manuscripts utilizing custom algorithms or software that are central to the research but not yet described in published literature, software must be made available to editors and reviewers. We strongly encourage code deposition in a community repository (e.g. GitHub). See the Nature Portfolio guidelines for submitting code & software for further information.

Data

Policy information about availability of data

All manuscripts must include a data availability statement. This statement should provide the following information, where applicable:

- Accession codes, unique identifiers, or web links for publicly available datasets
- A description of any restrictions on data availability
- For clinical datasets or third party data, please ensure that the statement adheres to our policy

The underlying code including MATLAB codes utilized to analyze spiking activities of ganglion cells is not publicly available but may be made available to qualified researchers on reasonable request from the corresponding author.

Research involving human participants, their data, or biological material

Policy information about studies with human participants or human data. See also policy information about sex, gender (identity/presentation), and sexual orientation and race, ethnicity and racism.

Reporting on sex and gender	N/A
Reporting on race, ethnicity, or other socially relevant groupings	N/A
Population characteristics	N/A
Recruitment	N/A
Ethics oversight	N/A

Note that full information on the approval of the study protocol must also be provided in the manuscript.

Field-specific reporting

Please select the one below that is the best fit for your research. If you are not sure, read the appropriate sections before making your selection.

☒ Life sciences ☐ Behavioural & social sciences ☐ Ecological, evolutionary & environmental sciences

For a reference copy of the document with all sections, see nature.com/documents/nr-reporting-summary-flat.pdf

Life sciences study design

All studies must disclose on these points even when the disclosure is negative.

Sample size	We clarified all sample sizes/stats throughout the main text. Also, those numbers of samples are widely considered to be sufficient.
Data exclusions	We did not exclude data.
Replication	To guarantee replication, we described all the details for all the experimental setup and methods, the animal strains and ages we used. also, we clearly described all the details regarding data collection and analyses.
Randomization	The thickness of photoreceptor layer was averaged from randomly selected areas in three retinas to determine the precise effect of EZH2 inhibitor-loaded hydrogels. After 2 weeks from the injection, physiological functions of retina tissues were examined at the level of single spike in each individual RGC, which was randomly chosen, using patch-clamping recordings.
Blinding	The experimenter was not blind to the identity of the animals.

Reporting for specific materials, systems and methods

We require information from authors about some types of materials, experimental systems and methods used in many studies. Here, indicate whether each material, system or method listed is relevant to your study. If you are not sure if a list item applies to your research, read the appropriate section before selecting a response.

Materials & experimental systems

n/a	Involved in the study	
☐	☒	Antibodies
☒	☐	Eukaryotic cell lines
☒	☐	Palaeontology and archaeology
☐	☒	Animals and other organisms
☒	☐	Clinical data
☒	☐	Dual use research of concern
☒	☐	Plants

Methods

n/a	Involved in the study	
☒	☐	ChIP-seq
☒	☐	Flow cytometry
☒	☐	MRI-based neuroimaging

Antibodies

Antibodies used

Tissues cryo-sectioned in 7 µm were stained with Anti-Iba1 antibody (#019-19741, Wako, Osaka, Japan), Anti-IL1β antibody (#sc-12742, Santa Cruz, Dallas, Texas, USA), Anti-TNF-α antibody (#ab183218, Abcam, Cambridge, Cambridgeshire, UK), Anti-CD68 antibody (#MCA1957T, Bio-Rad, Hercules, Contra Costa County, CA, USA), Anti-Cone arrestin antibody (#AB15282, Sigma-Aldrich, St.Louis, Missouri, USA), Anti-Cathepsin L antibody (#ab133641, Abcam, Cambridge, Cambridgeshire, UK), Anti-Cathepsin S antibody (#sc-271619, Santa Cruz, Dallas, Texas, USA), and Anti-Cathepsin B antibody (#ab214428, Abcam, Cambridge, Cambridgeshire, UK) as primary antibodies. The secondary antibodies were Donkey anti-Rabbit IgG antibody (#A21207, Invitrogen, Waltham, Massachusetts, USA) and Goat anti-Mouse IgG antibody (#A11001, Invitrogen, Waltham, Massachusetts, USA).

Validation

Data provided in the manuscript

Animals and other research organisms

Policy information about studies involving animals; ARRIVE guidelines recommended for reporting animal research, and Sex and Gender in Research

Laboratory animals

Wild-type mice (C57BL/6J strain) were purchased from Daehan BioLink (Eumseong, South Korea). Retinal degeneration 10 (rd10) mice were used as a retinitis pigmentosa (RP) model. We purchased breeding pairs of rd10 mice (B6.CXB1-Pde6brd10/J) from the Jackson Laboratory (Bar Harbor, ME) and a colony had been maintained in the KIST animal facility.

Wild animals

N/A

Reporting on sex

Both sexes were used.

Field-collected samples

N/A

Ethics oversight

The animal study was approved by Korea Institute of Science and Technology (KIST-5088-2022-05-077). All animal experiments were performed according to the institutional guidelines for the care and use of laboratory animals.

Note that full information on the approval of the study protocol must also be provided in the manuscript.
